# Nb-Dopant Changes the Regioselectivity of Proton-Coupled Electron Transfer in a Polyoxovanadate-Alkoxide

**DOI:** 10.1021/jacsau.5c00482

**Published:** 2025-06-12

**Authors:** Shannon E. Cooney, Thompson V. Marinho, S. Genevieve Duggan, Dominic Shiels, William W. Brennessel, Pere Miro, Ellen M. Matson

**Affiliations:** † Department of Chemistry, 6927 University of Rochester, Rochester, New York 14627, United States; ‡ Department of Chemistry, 4083 University of Iowa, Iowa City, Iowa 542240, United States; § Department of Chemistry, University of South Dakota, Vermillion, South Dakota 57069, United States

**Keywords:** proton-coupled electron transfer, bond dissociation free energy, polyoxovanadate, heterometal dopant, regioselectivity

## Abstract

The synthesis of a niobium­(V) substituted polyoxovanadate-alkoxide (NbPOV-alkoxide; [NbV_5_O_7_(OCH_3_)_12_]) is reported. Addition of 5,10-dihydrophenazine to [NbV_5_O_7_(OCH_3_)_12_] results in formation of the 2 H^+^/e^–^ reduced assembly, [NbV_5_O_6_(OH_2_)­(OCH_3_)_12_], via proton-coupled electron transfer. [NbV_5_O_6_(OH_2_)­(OCH_3_)_12_] has a bond dissociation free energy (BDFE­(O–H)_avg_) of 62.3 kcal mol^–1^, resembling that of its homometallic congener, [V_6_O_6_(OH_2_)­(OCH_3_)_12_] (BDFE­(O–H)_avg_ = 62.3 kcal mol^–1^). Single-crystal X-ray diffraction reveals that [NbV_5_O_6_(OH_2_)­(OCH_3_)_12_] exists as a mixture of two structural isomers, with the vanadium-aquo moiety formed in either the *trans*- or *cis*- positions relative to the Nb­(V) dopant. The formation of two regioisomers is a departure from prior observations of H-atom uptake at the surface of heterometal-doped polyoxovanadate-alkoxides, and is credited to distortions in intercluster metal oxygen bond lengths. Improved selectivity for the *trans*- isomer is achieved by decreasing the dielectric constant of the reaction solvent. Computational analysis predicts the preferential formation of *trans*-[NbV_5_O_6_(OH_2_)­(OCH_3_)_12_] in solvents with low dielectric constants as a result of changes to the dispersed charge across the assembly.

## Graphical abstract

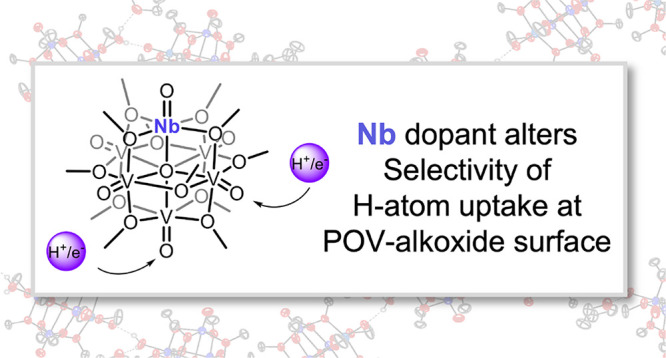

## Introduction

Transition metal oxides (MOx) have been proven competent for the mediation of a wide variety of multielectron transformations of small-molecule substrates.^1–6^ Investigations into these chemistries have revealed that small-molecule activation at the surface of MOx materials is often mediated by the transfer of proton–electron pairs (i.e., H-atom equivalents, H^·^).^7–9^ These reducing equivalents are stored in MOx materials as reactive hydroxy- or aquo- ligands. The thermochemistry of these bound H-atom equivalents dictates the range of substrates that can be (de)­hydrogenated by the MOx and is reliant on the coverage, size, and extent of reduction of the material.^8,9^

Considering that the reactivity of MOx is governed by the bond strength of surface-bound H-atoms (bond dissociation free energies, BDFEs), researchers are interested in understanding structure–function relationships that dictate the thermochemistry of these reactive moieties. One approach to modifying the surface chemistry of MOx assemblies is through the inclusion of heterometal dopants.^10^ Indeed, lattice dopants have been credited with modulating the reactivity of materials as a result of changes imparted to the electronic structure and charge allocation.^11–13^ Density functional theory (DFT) calculations predict that heterometal doping of MOx results in enhanced reducibility of the surface; a dopant often imparts increased lattice strain at neighboring sites by distorting the surrounding metal oxygen bond lengths, resulting in H-atom uptake at the adjacent nucleophilic sites.^14–17^ While DFT is a valuable tool for predicting the site of H-atom uptake, experimental support for the regioselectivity of H-atom uptake on heterometal-doped MOx remains challenging due to the inherent heterogeneity and scale of nanoscopic and extended materials (Figure 1).

**Figure 1:**
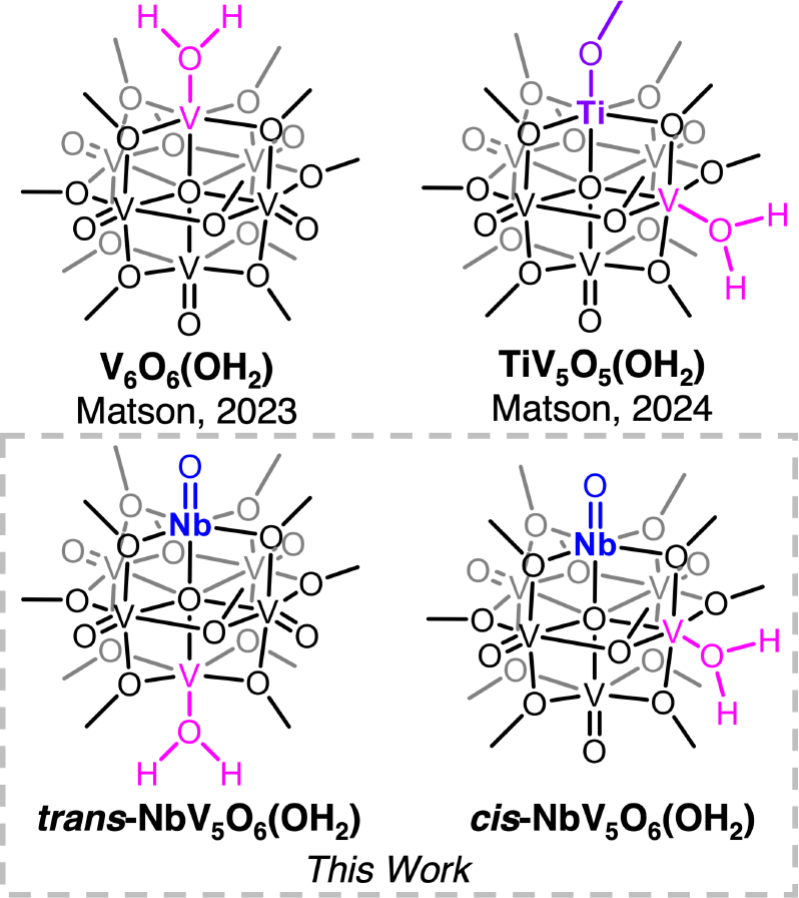
2H^+^/e^–^ reduced homometallic POV-alkoxide V_6_O_6_(OH_2_), and heterometal-doped POV-alkoxides discussed in this work.

To supplement DFT calculations, researchers have turned to the investigation of polyoxometalates as molecular analogues of MOx materials to achieve atomic-level insight into the surface reactivity of nanoscopic and extended solids.^18–20^ Polyoxometalates share a morphology similar to that of bulk materials (e.g., repeating units of bridging and terminal metal oxygen units), with the added benefit of a specific molecular structure and chemical formula. A subset of polyoxometalates used for this purpose by our research team is polyoxovanadate-alkoxides (POV-alkoxides).^21–24^ POV-alkoxides are vanadium-based assemblies consisting of terminal oxides and bridging methoxide ligands. These molecular species serve as good models for nanoscopic materials capped with organic ligands and possess a Robin and Day Class II electronic structure (i.e., partial delocalization of the metal ion charges), broadly resembling the semiconducting electronic structures of their extended MOx derivatives.^25,26^

Toward understanding the impact of cationic dopants on the electronic structure and reactivity of MOx, our group has reported a series of heterometal substituted POV-alkoxides, (MPOV-alkoxide; [(MX)­V_5_O_6_(OCH_3_)_12_], M = Fe, X = Br, Cl, ClO_4_, OTf, NO; M = Ti, Zr, Hf, X = OCH_3_; M = Ga, X = Cl).^27–29^ Inclusion of cationic dopants into the POV-alkoxide framework results in substantial changes to the physicochemical properties of the assembly. For example, upon substitution of a single vanadyl site with a Group IV metal dopant, the internal heterometal–oxygen bond length is shortened (M–O_c_ = 2.042(5)–2.153(2) Å; O_c_ = μ_6_-O^2–^), resulting in distortions to neighboring vanadium–oxygen bond lengths (average V–O_c_ = 2.343–2.365 Å).^29^ In addition, an anodic shift of the vanadium-based redox potentials is observed in comparison to its all-vanadium congener.^29^ Generally speaking, the reduction potentials of the vanadium-based redox events of MPOV-alkoxide clusters scale with the Lewis acidity of the heterometal dopant.^27^

Our team has been interested in understanding the impacts of cationic dopants on proton-coupled electron transfer (PCET) processes at the surface of POV-alkoxides. Recently, our group reported the generation of a 2 H^+^/e^–^ reduced TiPOV-alkoxide, [TiV_5_O_5_(OH_2_)­(OCH_3_)_13_] **TiV**_**5**_ **O**_**5**_ **(OH**_**2**_ **)**, via PCET, and demonstrated that H-atom equivalents are installed at a vanadyl ion positioned *cis-* to the Ti­(IV) ion (Figure 1). Exclusive reactivity at a vanadyl site adjacent to the heterometal dopant is notable, as it mimics the regioselectivity of H-atom uptake predicted from DFT studies of doped MOx.^30^ In addition, the isovalent Ti­(IV) ion was found to have a similar electronic impact as a V­(IV) on the strength of bound H-atoms at the aquo ligand, suggesting charge state distributions of metal ions, and not overall d-electron counts, are the principal determinant of the thermochemistry of surface O–H bonds.^30,31^

Herein, we report the structural and electronic effects of the incorporation of a single Nb­(V) dopant in a Lindqvist-type POV-alkoxide. First, the syntheses of two redoxmers of the Nb-doped POV-alkoxides, [NbV_5_O_7_(OCH_3_)_12_]^n^ (*n* = 1–, 0), are described. Subsequent investigations into the reactivity of the Nb-substituted assembly, [NbV_5_O_7_(OCH_3_)_12_], with 5,10-dihydrophenazine reveal the addition of two H-atoms to a single vanadyl site of the assembly, resulting in the formation of an aquo adduct of the reduced, protonated species, [NbV_5_O_6_(OH_2_)­(OCH_3_)_12_]. Intriguingly, structural characterization of the NbPOV-alkoxide shows H-atom uptake at vanadium sites positioned both *trans*- and *cis*- to the heterometal dopant; this observation is distinct from the *cis*-selectivity reported previously by our group for the Ti-doped POV-alkoxide. Structural data suggest that perturbations in lattice metal–oxygen bond lengths internal to the cluster (i.e., lattice) are major contributors to the observed change in regioselectivity of H-atom uptake. Computational analysis indicates a slight thermodynamic preference for H-atom uptake at the vanadyl *trans*- to the dopant that is independent of BDFE­(O–H)_avg_. The selectivity for *cis-* vs *trans-* isomers can be perturbed by changing the dielectric constant of the reaction solvent, with lower polarity solvents stabilizing the *trans-* isomer (relative to the *cis-*). Our results reveal the implications of dopant identity on the selectivity of H-atom uptake and relevance for the design of MOx catalysts for selective (de)­hydrogenation chemistries.

## Results and Discussion

### Synthesis and Characterization of a NbPOV-Alkoxide

Initial attempts to synthesize a NbPOV-alkoxide mirrored approaches reported previously for the Group­(IV) transition metal substituted assemblies.^32^ Nb_2_(OEt)_10_, VO­(OMe)_3_, and [^n^Bu_4_N]­[BH_4_] were added together in methanol (1:5:1 ratio, respectively); the green solution was placed in the Teflon liner of a 25 mL stainless steel autoclave. The reaction was sealed and heated to 150 °C for 24 h. After cooling, the resultant brown solution was analyzed by electrospray ionization mass spectrometry (ESI-MS); one major signal was observed in the negative mode at *m*/*z* = 832, consistent with the anticipated mass and isotope splitting of the desired product, [NbV_5_O_7_(OMe)_12_]^1–^, (**NbV**_**5**_ **O**_**7**_^**1–**^; Figure S1). However, a large number of smaller signals are also observed, some of which correspond to undesired vanadium oxide clusters (V_6_O_7+x_(OMe)_12–*x*_); *x* = 0, 1, 2, 3).^33,34^ The abundance of these impurities indicates that further modifications of the reaction procedure are necessary.

Pure samples of **NbV**_**5**_ **O**_**7**_^**1–**^ were obtained upon modifying the reaction conditions by increasing the Nb-to-V precursor ratio to 3:5 (Scheme 1; Figure S2). Isolation of the Nb-doped assembly as a light-green powder is possible following workup (see the Experimental Section for details). Purity of the product was confirmed by ESI-MS, elemental analysis, ^1^H NMR, and infrared spectroscopies (Figures S3–S5). The ESI-MS spectrum of **NbV**_**5**_ **O**_**7**_^**1–**^ shows a major peak at *m*/*z* = 832 and a minor peak at *m*/*z* = 846, which corresponds to the mass of the [NbV_5_O_7_(OCH_2_CH_3_)­(OCH_3_)_11_]^1–^ ion. The ^1^H NMR spectrum of **NbV**_**5**_ **O**_**7**_^**1–**^ possesses three paramagnetically shifted and broadened resonances (δ = 11.3, 23.9, 27.1 ppm), along with signals in the diamagnetic region that are assigned to the protons of the [^*n*^Bu_4_N]^1+^ countercation (δ = 0.97, 1.33, 1.59, 3.07 ppm). The three paramagnetic signals are consistent with the expected number of resonances for *pseudo*-C_4v_ symmetric NbPOV-alkoxide.

**Scheme 1:**
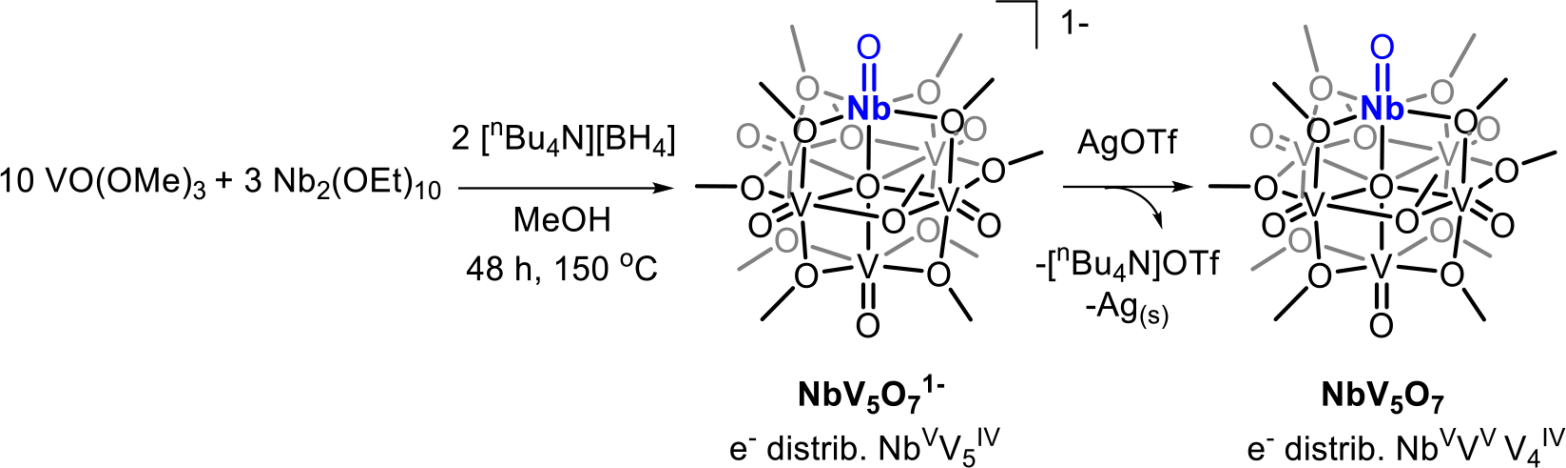
Synthesis of Nb-Doped POV-Alkoxide Clusters

The electronic structure of **NbV**_**5**_ **O**_**7**_^**1–**^ was further interrogated by electronic absorption spectroscopy (EAS; Figure 2). Two, low-intensity absorptions are observed in the visible/near-infrared region of the spectrum, 970 nm (ε = 135 M^–1^ cm^–1^) and 625 nm (ε = 67 M^–1^ cm^–1^). These transitions resemble the line shape and intensity of spectra reported for reduced, isovalent POV-alkoxide , namely, [V_6_O_7_(OCH_3_)_12_]^2–^ (oxidation state distrib. = V^IV^_6_)^25^ and [MV_5_O_6_(OCH_3_)_13_]^1–^ (M = Zr, Hf; oxidation state distrib. = M^IV^V^IV^_5_),^29^ and are assigned as V^IV^ *d* → *d* transitions. This observation suggests that the five vanadium centers of **NbV**_**5**_ **O**_**7**_^**1–**^ are all in the 4+ oxidation state. In this scenario, charge balance would necessitate Nb to be in the 5+ oxidation state, resulting in an oxidation state distribution of Nb^V^V^IV^_5_ for **NbV**_**5**_ **O**_**7**_^**1–**^. We note that an additional absorption is observed in the spectrum of **NbV**_**5**_ **O**_**7**_^**1–**^ at 420 nm (ε = 420 M^–1^ cm^–1^). This transition resembles a band present in the TiPOV-alkoxide, [TiV_5_O_6_(OCH_3_)_13_]^1–^, assigned as an intervalence charge transfer (IVCT) event between a *d* ^1^ vanadium ion and the *d* ^0^ titanium dopant.^29^ The presence of a similar transition for **NbV**_**5**_ **O**_**7**_^**1–**^ implies that exchange of electron density between vanadium (*d* ^1^) and niobium (*d* ^0^) is similarly possible in this heterometallic assembly.

**Figure 2:**
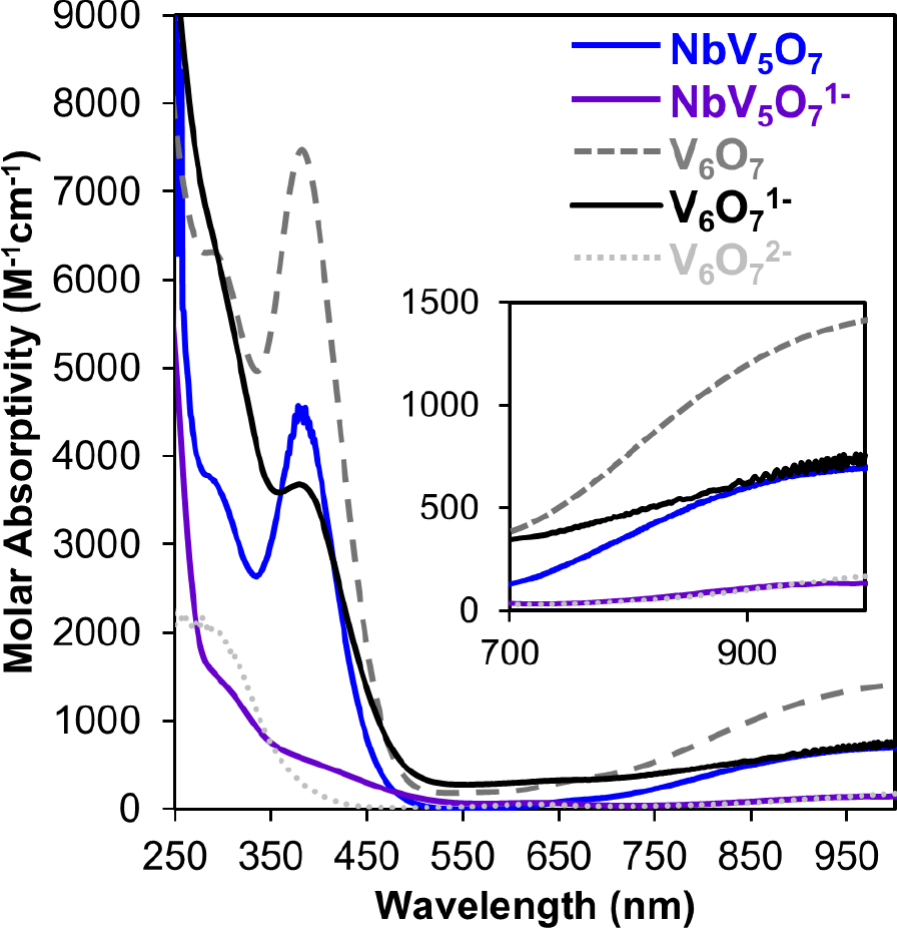
EAS of **NbV**_**5**_ **O**_**7**_ and **V**_**6**_ **O**_**7**_ clusters in various charge states in acetonitrile collected at room temperature.

The electrochemical profile of **NbV**_**5**_ **O**_**7**_^**1–**^ was measured in acetonitrile via cyclic voltammetry (CV; 100 mV s^1–^, 0.1 M [^*n*^Bu_4_N]­[PF_6_] used as the supporting electrolyte, Figure 3 and Table 1). The open circuit potential (OCP) of the Nb-doped POV-alkoxide is found at −0.51 V (vs Fc^+/0^); three oxidation events are observed at −0.25, 0.31, and 0.82 V (vs Fc^+/0^), closely resembling the oxidation events observed in the CV of the all-vanadium assembly, [V_6_O_7_(OCH_3_)_12_]^1–^.^25,26^ When the electrochemical profile of **NbV**_**5**_ **O**_**7**_^**1–**^ is compared to that of **V**_**6**_ **O**_**7**_^**1–**^, the reduction process observed at −0.78 V vs Fc^+/0^ for **V**_**6**_ **O**_**7**_^**1–**^ is conspicuously absent. We attribute this to the increased overpotential required for the reduction of the Nb^V^ dopant. Indeed, expanding the window of the electrochemical experiment to more reducing potentials results in the observation of an additional reduction event at −2.37 V vs Fc^+/0^, which we assign as the Nb^V/IV^ couple (Figure S6).^35,36^ The reduction event assigned to the Nb-dopant is considerably less reversible than the vanadium-based redox events; the peak-to-peak separation of the anodic and cathodic events (Δ*E*_p_) deviates significantly from the ideal value of 57 mV (Δ*E*_p_ = 210 mV).^37^ We note that historically, Nb-based redox chemistry in polyoxometalates has been exclusively studied in aqueous environments due to the high charges associated with the polyoxometalate, rendering resolution of Nb-based events challenging.^35,36^ However, by switching to a nonaqueous solvent, a larger potential window can be investigated, allowing for the resolution of Nb-based reduction events.

**Figure 3:**
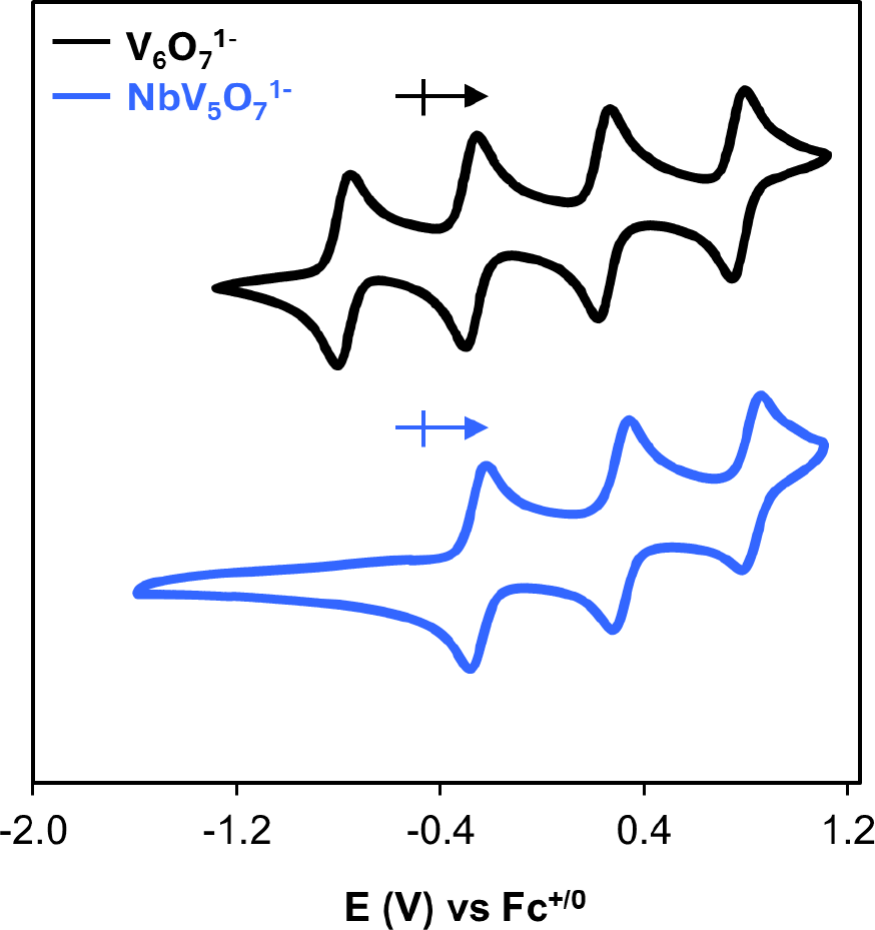
Cyclic voltammograms of **V**_**6**_ **O**_**7**_^**1–**^ (black) and **NbV**_**5**_ **O**_**7**_^**1–**^ (blue) collected in acetonitrile with 0.1 M [^*n*^Bu_4_N]­[PF_6_] as the supporting electrolyte.

**Table 1:** Electrochemical Parameters Measured for **V**_**6**_ **O**_**7**_ and **NbV**_**5**_ **O**_**7**_^**1–**^

	V_6_O_7_	NbV_5_O_7_
redox event	M = V	M = Nb
[M^V^V^IV^_5_]^1–^ + e^–^ → [M^IV^V^IV^_5_]^2–^	–0.78	–2.37
[M^V^V^V^V^IV^_4_]^0^ + e^–^ → [M^V^V^IV^_5_]^1–^	–0.28	–0.25
[M^V^V^V^_2_V^IV^_3_]^1+^ + e^–^ → [M^V^V^V^V^IV^_4_]^0^	+0.25	+0.31
[M^V^V^V^_3_V^IV^_2_]^2+^ + e^–^ → [M^V^V^V^_2_V^IV^_3_]^1+^	+0.79	+0.82

### H Atom Uptake on Nb-Doped POV-Alkoxide Clusters

Our research group is interested in understanding H-atom uptake and transfer at the surface of POV-alkoxides.^38^ In our prior studies, the presence of a formal V^V^ center in the Lindqvist core has been established as essential for “turning-on” H-atom uptake at the surface of the assembly; clusters where all-vanadium centers are in the 4+ oxidation state (e.g., **V**_**6**_ **O**_**7**_^**2–**^, [TiV_5_O_6_(OCH_3_)_13_]^1–^) are unreactive with H-atom transfer reagents.^39^ Above, we establish the oxidation state distribution of metal ions in **NbV**_**5**_ **O**_**7**_^**1–**^ as Nb^V^V^IV^_5_; while the cluster does not have a V^V^ center, we hypothesized that H-atom uptake might be possible at the heterometal ion (Nb^V^ = *d* ^0^) or, alternatively, at a vanadium center following intracluster electron transfer (V^IV^ → Nb^V^). Reduction of **NbV**_**5**_ **O**_**7**_^**1–**^ with a potent H-atom transfer reagent, dihydrophenazine (H_2_Phen; BDFE­(N–H)_avg_ = 58.7 kcal mol^–1^ in acetonitrile^40^), was attempted; however, after prolonged reaction periods at elevated temperatures, no reaction was observed (Figure S7).

In an attempt to promote the reactivity of the Nb-doped POV-alkoxide with H-atom equivalents, **NbV**_**5**_ **O**_**7**_^**1–**^ was oxidized with silver trifluoromethanesulfonate (AgOTf) in dichloromethane to generate [NbV_5_O_7_(OCH_3_)_12_] (**NbV**_**5**_ **O**_**7**_, Scheme 1). Following workup, the product was characterized by ^1^H NMR spectroscopy (see the Experimental Section for details). Three paramagnetically shifted and broadened signals are observed at 39.1, 16.6, and 9.9 ppm, distinct resonances from those of the starting material (Figure S8). Inspection of the diamagnetic region of the spectrum revealed the loss of signals corresponding to the countercation, [^*n*^Bu_4_N]^1+^, consistent with oxidation of the assembly. Further evidence of successful oxidation was obtained by measurement of the OCP of the product, which was found to shift anodically over the first V^V^/V^IV^ couple (OCP = −0.08 V vs Fc^0/+^) (Figure S9). Confirmation of the generation of a V^V^ within **NbV**_**5**_ **O**_**7**_ was obtained through EAS; an intense ligand-to-metal charge transfer band was observed (387 nm M^–1^ cm^–1^), as well as a V^V^/V^IV^ IVCT process (1036 M^–1^ cm^–1^) (Figure 2). These features are indicative of mixed-valent (V^V^/V^IV^) POV-alkoxides, consistent with an oxidation state distribution with the neutral Nb-doped POV-alkoxide of Nb^V^V^V^V^IV^_4_.^33,41,28^ Characterization of **NbV**_**5**_ **O**_**7**_ was also performed through single-crystal X-ray diffraction (Figure S10 and Table S1) and combustion analysis (see the Experimental Section for details).

With the oxidized NbPOV-alkoxide in hand, we next investigated its reactivity with H-atom transfer reagents. When H_2_Phen is added to **NbV**_**5**_ **O**_**7**_, a rapid color change from green to orange is observed. Analysis of the crude reaction mixture by ^1^H NMR spectroscopy reveals full conversion of the starting materials, H_2_Phen and **NbV**_**5**_ **O**_**7**_, to the dehydrogenated substrate, phenazine (Phen), and a new product with paramagnetically shifted and broadened resonances ranging from −20 to +30 ppm, tentatively assigned as [NbV_5_O_6_(OH_2_)­(OCH_3_)_12_] (**NbV**_**5**_ **O**_**6**_ **(OH**_**2**_ **)**; Figure S11).

Unambiguous confirmation of H-atom uptake at the surface of the NbPOV-alkoxide was obtained through single-crystal X-ray diffraction. Crystals suitable for X-ray analysis of **NbV**_**5**_ **O**_**6**_ **(OH**_**2**_ **)** were grown by sequential vapor diffusion of diethyl ether and then pentane into a concentrated solution of the product in tetrahydrofuran (THF; Figure 4 and Table S2). Preliminary crystallographic data suggests that the dominant location of the aquo is positioned *trans-* to the Nb-dopant (*trans-* **NbV**_**5**_ **O**_**6**_ **(OH**_**2**_ **)**); however, structural refinement of the disorder suggests that an isomer exists with the aquo ligand in the *cis*- position in a 3:1 *trans-*/*cis-* ratio (*cis-* **NbV**_**5**_ **O**_**6**_ **(OH**_**2**_ **)**; Figure 4b and Scheme 2). BVS calculations suggest that in both isomers the vanadium site bearing the aquo ligand is a localized V^III^ ion, consistent with previously reported reduced POV-alkoxides (Table S3 and Figure S12).^25,31,42–47^

**Scheme 2:**
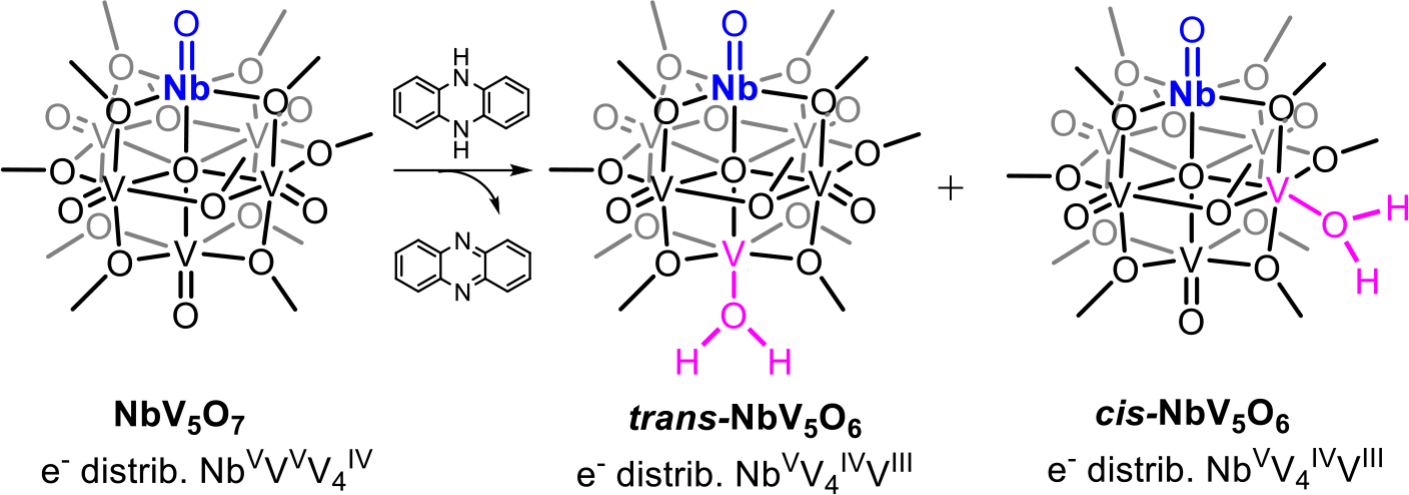
H Atom Uptake at the Surface of **NbV**_**5**_ **O**_**7**_ Resulting in the Formation of **NbV**_**5**_ **O**_**6**_ **(OH**_**2**_ **)**

**Figure 4:**
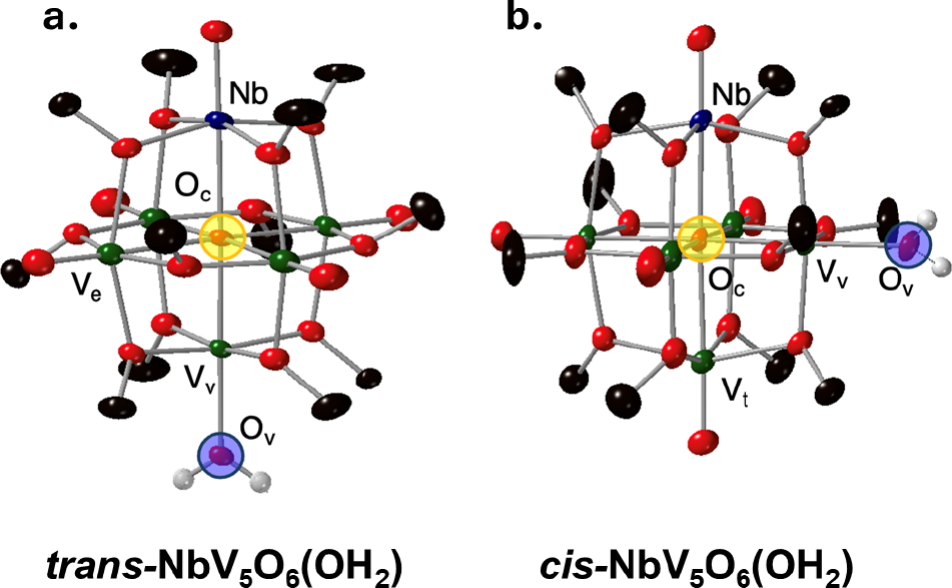
X-ray structures of (a) *trans* **-NbV**_**5**_ **O**_**6**_ **(OH**_**2**_ **)** and (b) *cis* **-NbV**_**5**_ **O**_**6**_ **(OH**_**2**_ **)**. Oxygen atoms are labeled as red, carbon as black, select hydrogen atoms as white, vanadium atoms as green, and niobium as dark blue. *Trans*- and *cis*- aquo positions are highlighted with blue circles.

The observation of two structural isomers of **NbV**_**5**_ **O**_**6**_ **(OH**_**2**_ **)**, where the 2 H^+^/e^–^ reduction occurs at the *trans*- or *cis-* vanadyl with respect to the Nb­(V), is notably different from the selectivity observed for **TiV**_**5**_ **O**_**5**_ **(OH**_**2**_ **)**, previously characterized by our group.^30^ In the case of the Ti-doped assembly, H-atom uptake occurs exclusively at the *cis*- position. We justify this variance in reactivity through structural discrepancies across the Ti- and Nb-doped assemblies (Table S4). In the case of the TiPOV-alkoxide cluster, we note that the titanium to central metal–oxygen (O_c_) bond length is quite short (Ti–O_c_ = 2.030(5) Å).^29,30^ This leads to a lengthening of the V–O_c_ bond (2.500(5) Å) *trans* to the Ti dopant, decreasing the reducibility of the chemically distinct vanadyl site. Indeed, our previous work has shown that formation of a V^III^–OH_2_ is paired with the formation of a short V^III^–O_c_ bond (e.g., 2.1158(16) Å for [V_6_O_6_(OMe)_12_(OH_2_)]), likely offsetting the lengthening of the terminal V–O bond during H-atom uptake.^30,31^ It is therefore reasonable to expect that lengthening of the V–O_c_ bond of the vanadyl group *trans*- to the Ti dopant prevents H-atom uptake at this position. However, doping the POV-alkoxide with Nb is not expected to lead to the same lattice perturbations. The comparatively large ionic radius of Nb (vs the Ti dopant) and the presence of a Nb=O moiety *trans*- to the Nb–O_c_ group necessitate a longer Nb–O_c_ bond, which is likely to be similar to the other V–O_c_ bond lengths in the assembly.^48,49^ This leads to a more symmetrical lattice, with the V–O_c_ bonds of the *trans*- and *cis-* vanadyls being similar in length, therefore turning on reactivity with H-atom equivalents at both sites.

Benchmarking experiments were performed with HAT reagents of decreasing potency to probe the strength of the O–H bonds of **NbV**_**5**_ **O**_**6**_ **(OH**_**2**_ **)**. As introduced previously, **NbV**_**5**_ **O**_**7**_ reacts fully with H_2_Phen. Similar conversion to **NbV**_**5**_ **O**_**6**_ **(OH**_**2**_ **)** is observed upon addition of hydrazobenzene (H_2_Azo; BDFE­(N–H)_avg_ = 60.9 kcal mol^–1^)^40^ to **NbV**_**5**_ **O**_**7**_ (Figure S13). However, addition of 1,4-dihydroxynaphthalene (H_2_NQ; BDFE­(O–H)_avg_ = 62.6 kcal mol^–1^)^40^ results in only partial conversion to naphthoquinone, consistent with establishing an equilibrium between **NbV**_**5**_ **O**_**7**_ and H_2_NQ (Figure S14). We note that these investigations were performed on mixtures of *cis-* and *trans-* isomers of the reduced assembly; however, inspection of the product distribution over the course of equilibration indicates the ratios of isomers remain constant throughout the experiment. This data suggests that the O–H bond of *cis-* **NbV**_**5**_ **O**_**6**_ **(OH**_**2**_ **)** and *trans-* **NbV**_**5**_ **O**_**6**_ **(OH**_**2**_ **)** can be considered comparable (vide infra). The equilibrium is used to calculate the BDFE­(O–H)_avg_ of **NbV**_**5**_ **O**_**6**_ **(OH**_**2**_ **)** (Figures S15, S16 and Table S5), using a modified version of the Bordwell equation popularized by Mayer and co-workers.^8^ The BDFE­(O–H)_avg_ of H-atoms bound to **NbV**_**5**_ **O**_**6**_ **(OH**_**2**_ **)** is found to be 62.3 ± 0.1 kcal mol^–1^ (see the Experimental Section for details pertaining to calculation). The moderately weak BDFE­(O–H)_avg_ of **NbV**_**5**_ **O**_**6**_ **(OH**_**2**_ **)** is demonstrated by its reactivity in the presence of oxygen. Analysis of the sample by EAS reveals that **NbV**_**5**_ **O**_**6**_ **(OH**_**2**_ **)** is oxidized to **NbV**_**5**_ **O**_**7**_ when exposed to air over the course of 6 h (Figure S17). These results mirror previous findings from our group, which show that a POV-alkoxide with a V^III^–OH_2_ moiety, [V_6_O_6_(OH_2_)­(OCH_3_)_12_]^1–^, is capable of facilitating the stochiometric reduction of O_2_ via a PCET mechanism.^50^

Quantification of the thermochemistry of O–H bonds at the vanadium-aquo sites (e.g., BDFE­(O–H)_avg_ of **NbV**_**5**_ **O**_**6**_ **(OH**_**2**_ **)**) allows for studying the effects of the electronic structure (e.g., metal ion and valency) on H-atom uptake at the surface of the NbPOV-alkoxide. The BDFE­(O–H)_avg_ value of **NbV**_**5**_ **O**_**6**_ **(OH**_**2**_ **)** is first compared to that of its all-vanadium congener, [V_6_O_6_(OH_2_)­(OCH_3_)_12_] (**V**_**6**_ **O**_**6**_ **(OH**_**2**_ **)**). The experimentally determined BDFE­(O–H)_avg_ of the homometallic POV-alkoxide, **V**_**6**_ **O**_**6**_ **(OH**_**2**_ **)** (62.3 ± 0.1 kcal mol^–1^), is statistically equivalent to that of **NbV**_**5**_ **O**_**6**_ **(OH**_**2**_ **)**. This result is consistent with a prior work that suggests the BDFE­(O–H) of aquo ligands at the surface of reduced POV-alkoxides is most sensitive to oxidation state distributions of the constituent metal ions.^39^ The electronic structure of **NbV**_**5**_ **O**_**6**_ **(OH**_**2**_ **)** (ox. state distrib. = Nb^V^V^III^V^IV^_4_) is similar to that of V_6_O_6_(OH_2_)­(OCH_3_)_12_ (ox. state distrib. = V^III^V^IV^_4_V^V^), where the *d* ^0^ Nb­(V) dopant is substituted for the *d* ^0^ V­(V) center. In contrast, the BDFE­(O–H)_avg_ value of **NbV**_**5**_ **O**_**6**_ **(OH**_**2**_ **)** is 2 kcal mol^–1^ stronger than the reported value for **TiV**_**5**_ **O**_**5**_ **(OH**_**2**_ **)** (60.1 ± 0.1 kcal mol^–1^).^30^ The TiPOV-alkoxide possesses an oxidation state distribution of Ti^IV^V^III^V^IV^_4_; while the Ti center is also a *d* ^0^ ion, the *charge* of this metal is reduced in comparison to that of its Group­(V) congener. This difference changes both the charge of the overall assembly and inductive effects within the cluster, translating to a higher BDFE­(O–H)_avg_ for **NbV**_**5**_ **O**_**6**_ **(OH**_**2**_ **).**

### Modifying Regioselectivity of Isomer Formation

After detecting the formation of the *cis*- and *trans*- isomers in the crystal structure of **NbV**_**5**_ **O**_**6**_ **(OH**_**2**_ **)**, we sought to isolate the individual species by varying reaction conditions to increase the selectivity toward a single product. Initially, we hypothesized that regioselectivity might be a function of a thermodynamic preference for the *cis-* or *trans-* isomer. Addition of H_2_Phen to **NbV**_**5**_ **O**_**7**_ over a range of temperatures (−30 to +50 °C) results in no major differences in the peak distribution by ^1^H NMR spectroscopy (Figure S18). Similarly, varying the driving force of the reaction by altering the substrate added (e.g., H_2_Phen vs H_2_Azo) has no impact on product distribution. Indeed, comparison of product distributions following the reaction of **NbV**_**5**_ **O**_**7**_ with either H_2_Phen (BDFE­(N–H) = 59 kcal mol^–1^) or H_2_Azo (60 kcal mol^–1^) in acetonitrile (Figures S12 and S13) reveals no major differences in amounts of ***cis-*** vs *trans-* **NbV**_**5**_ **O**_**6**_ **(OH**_**2**_ **)**. This implies that there are no substantial changes in the driving force for the formation of the new O–H bonds on either of the two isomers of **NbV**_**5**_ **O**_**6**_ **(OH**_**2**_ **)**. Finally, no change in the *cis-* vs *trans*- product distribution was observed when the reaction was monitored over extended periods under equilibrium conditions. The experimental results are consistent with DFT calculations performed by our team that predict similar BDFE­(O–H)_avg_ values for the two regioisomers of **NbV**_**5**_ **O**_**6**_ **(OH**_**2**_ **)** (see the Experimental Section for more details). Thermochemical values were obtained from the optimized structures of the relevant reduced NbPOV-alkoxides *cis* **-NbV**_**5**_ **O**_**6**_ **(OH**_**2**_ **)** and *trans* **-NbV**_**5**_ **O**_**6**_ **(OH**_**2**_ **)**. The BDFE­(O–H)_avg_ values are determined from the first (V^IV^–OH) and second (V^III^–OH_2_) H-atom transfers to **NbV**_**5**_ **O**_**7**_. The BDFE­(O–H)_avg_ values of *cis* **-NbV**_**5**_ **O**_**6**_ **(OH**_**2**_ **)** and *trans* **-NbV**_**5**_ **O**_**6**_ **(OH**_**2**_ **)** were calculated to be 66.8 and 68.0 kcal mol^–1^ in THF, respectively (note that the difference in BDFE is within the error of the calculation performed).

With the thermochemical control eliminated as a determinant of selectivity of PCET, we next examined kinetic elements of the synthesis of **NbV**_**5**_ **O**_**6**_ **(OH**_**2**_ **)** that may yield improved isomer selectivity. PCET reaction kinetics (e.g., rate, mechanism) have been shown to be sensitive to solvent identity; as such, we opted to change the reaction solvent in an attempt to favor the formation of a single isomer.^51–54^ Indeed, by switching from acetonitrile as a solvent to THF, proton resonances at 30.33, 14.01, and −10.44 ppm increase in intensity (Figure 5). This 3-peak pattern follows the expected symmetry of the *trans*- isomer, which retains the symmetry of the parent cluster **NbV**_**5**_ **O**_**7**_ of C_4v_ (aquo ligand is formed at a vanadyl site coincident with the C_4_ axis of the assembly). Interested in improving the selectivity for *trans* **-NbV**_**5**_ **O**_**6**_ **(OH**_**2**_ **)**, we screened the reaction in different solvents with decreasing dielectric constants: acetonitrile, dichloromethane, THF, and diethyl ether (Figure 5). By decreasing the dielectric constant, we observe an increased preference for the formation of the *trans* **-NbV**_**5**_ **O**_**6**_ **(OH**_**2**_ **)** assembly, up to a 5:1 ratio (Table 2, determined in situ via integration of the paramagnetic ^1^H NMR).

**Figure 5:**
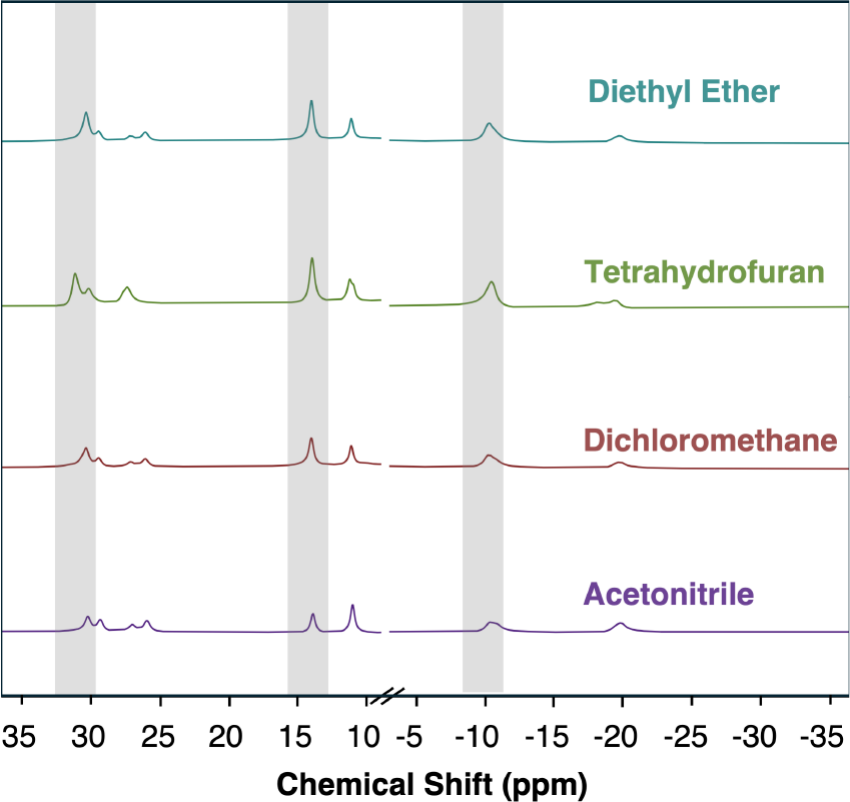
Comparison of crude reaction mixtures of **NbV**_**5**_ **O**_**7**_ + H_2_Phen in various solvents. Peaks corresponding to *trans* **-NbV**_**5**_ **O**_**6**_ are highlighted in gray^1^ (21 °C).

**Table 2:** Ratio of *trans*-/*cis*- Isomer Determined In Situ

	ε	ratio (*trans-/cis-*)
acetonitrile	21.0	2:1
tetrahydrofuran	7.6	3:1
dichloromethane	8.9	3:1
diethyl ether	3.1	5:1

Our initial observations indicate that decreasing the dielectric constant of the solvent favors the formation of *trans* **-NbV**_**5**_ **O**_**6**_ **(OH**_**2**_ **)**. Plotting the ratio of *trans*-/*cis*- isomers vs ε reveals a linear correlation with an excellent fit (Figure S19, *R* ^2^ = 0.99). Other solvent parameters were screened to elucidate possible origins of the isomer-solvent dependence: acceptor number, donor number, viscosity, Reichardt’s empirical solvent polarity parameter (E_T_(30)), and refractive index (Figure S20). E_T_(30) shows a moderate correlation (*R* ^2^ = 0.83); we hypothesize that this connection is a result of E_T_(30) accounting for the polarity of the solvent. All other parameters screened showed no correlation (0 ≤ *R* ^2^ ≤ 0.4). Dielectric constant (ε) is also a measure of solvent polarity, which is quantified by measuring a solvent’s ability to insulate charge.^55^ This manifests as high ε solvents more efficiently solvating polar molecules. Thus, due to the strong correlation of the *trans-/cis-* ratio and ε, we hypothesize that there may be substantial differences in the polarity of *trans* **-/** *cis* **-NbV**_**5**_ **O**_**6**_ **(OH**_**2**_ **)**.

To quantify the polarity of the isomers of **NbV**_**5**_ **O**_**6**_ **(OH**_**2**_ **)**, DFT calculations were employed. The isomer dipole moments for the *trans*- and *cis-* isomers (μ_*trans*_ and μ_*cis*_, respectively) were calculated in diethyl ether. *Cis-* **NbV**_**5**_ **O**_**6**_ **(OH**_**2**_ **)** has a μ*_cis_* of 12.6 debye compared to a μ_*trans*_ of 7.8 debye for *trans-* **NbV**_**5**_ **O**_**6**_ **(OH**_**2**_ **)**, revealing that *trans*- is less polar than the *cis*- isomer. To visualize the polarities of the reduced NbPOV-alkoxides, the electrostatic potential maps are presented for both *trans-* and *cis-* isomers (Figure 6a,b). For the *cis*- isomer, there is an increase in polarity imparted by the localization of the negative charge at the V^III^–OH_2_ in the equatorial plane to the Nb=O. In contrast, with the V^III^–OH_2_ moiety positioned in the C_4v_ axis of the *trans*- isomer, the negative charges are more dispersed, resulting in a lower μ. While differences in the polarity are noteworthy, an intriguing trend emerges from investigating the free energies of the isomers across solvents of various ε. The optimized structures reveal that as ε is decreased, the difference in Gibbs free energy of the two isomers increases (i.e., *trans*- is more energetically favored than *cis*-). To visualize this effect, the difference in the Gibbs free energy of the isomers (Δ*G*_isomer_ **=** |*G_cis_* – G_*trans*_|) (Figure 6c) is plotted as a function of ε^–1^. A strong correlation between Δ*G*_isomer_ and ε^–1^ is observed, such that *trans*- is favored in low ε, mirroring our experimental findings. Based on the computational results presented here, we propose that the improved yield of the *trans*- isomer is a result of a higher driving force provided by the improved stability of the product in a low ε solvent. We note that the energy differences between the isomers are small, ∼3 kcal mol^–1^ in diethyl ether, and this likely accounts for the observed mixtures of isomers across the range of solvents investigated. In addition, solvent interactions are likely present in the transition state, affecting activation barriers; however, calculations on those intermediates could have a multireference character, placing them beyond the scope of this study. Consequently, we cannot eliminate the possibility of kinetic solvent influences.

**Figure 6:**
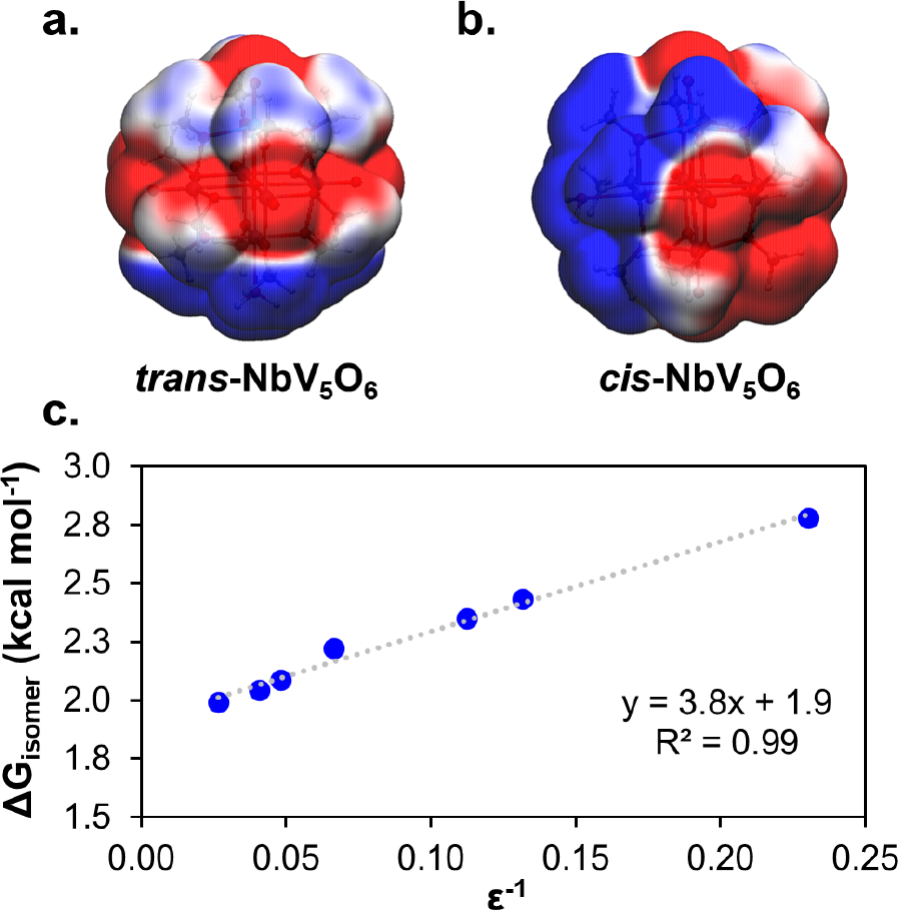
Electrostatic potential maps of (a) *trans-* **NbV**_**5**_ **O**_**6**_ and (b) *cis-* **NbV**_**5**_ **O**_**6**_. Color code: blue, negative charge; red, positive charge. Isovalue: 0.001. (c) Plot of difference of the isomer free energy (Δ*G*_isomer_) as a function of ε^–1^.

## Conclusions

In this study, we report the synthesis of Nb-doped POV-alkoxides, **NbV**_**5**_ **O**_**7**_^**n**^, *n* = 1–, 0, and the 2 H^+^/e^–^ reduced assembly, **NbV**_**5**_ **O**_**6**_ **(OH**_**2**_ **)**. The BDFE­(O–H)_avg_ of **NbV**_**5**_ **O**_**6**_ **(OH**_**2**_ **)** is found to be 62.3 ± 0.1 kcal mol^–1^, which mirrors the BDFE­(OH)_avg_ reported as its homometallic analogue, V_6_O_6_(OH_2_)­(OCH_3_)_12_. Notably, the BDFE­(OH)_avg_ value of **NbV**_**5**_ **O**_**6**_ **(OH**_**2**_ **)** is ∼2 kcal mol^–1^ stronger than the previously reported Group (IV) substituted POV-alkoxide, **TiV**_**5**_ **O**_**5**_ **(OH**_**2**_ **)**. H-atom uptake at **NbV**_**5**_ **O**_**7**_ results in the generation of two structural isomers of **NbV**_**5**_ **O**_**6**_ **(OH**_**2**_ **)** (i.e., *cis-* and *trans-*isomers). Formation of two isomers occurs as a result of the elongation of structural perturbations within the cluster core as a result of the niobium dopant. Specifically, the elongation of the Nb–O_c_ bond, in comparison to that of the dopant–O_c_ distance in its titanium homologue, activates the *trans-*vanadyl site for H-atom uptake. The distribution of products of **NbV**_**5**_ **O**_**6**_ **(OH**_**2**_ **)** is shown to be dependent on the solvent dielectric constant; DFT calculations reveal that the origin of this effect is a consequence of differences in charge allocation across the POV-assembly for the aquo moiety in the *trans*- vs *cis*- position.

As described above, the results presented in this work suggest that regioselectivity of H-atom uptake in heterometal-doped POV-alkoxides is determined by distortions of intercluster metal–oxygen bond lengths. As POV-alkoxides and other polyoxometalates have been studied as molecular models for bulk metal oxide materials, these findings provide new insights into the role of heterometal dopants in the location of H-atom uptake (i.e., reduction) at metal oxide surfaces. The ability to control the regioselectivity of H-atom uptake on doped MOx materials may have implications for the activity of MOx-mediated catalysis. If *trans-* active sites on POV-alkoxides are akin to internal H-atom uptake (i.e., proton-coupled electron intercalation), then inclusion of large transition metal dopants may be beneficial to modify intercalative reactivity. In contrast, our results suggest that the activation of surface O-atoms (i.e., H-atom uptake at the *cis*- position), which is a key intermediate in MOx-mediated transformations, is more directly influenced by the integration of smaller transition metal ions.^56^

## Experimental Section

### General Considerations

All manipulations were carried out in the absence of water and oxygen using standard Schlenk techniques or in a UniLab MBraun inert atmosphere drybox under a dinitrogen atmosphere. All glassware was oven-dried for a minimum of 4 h and cooled in an evacuated antechamber prior to use in the drybox. Anhydrous methanol was purchased from Sigma-Aldrich and stored over activated 4 Å molecular sieves, purchased from Fisher Scientific. All other solvents were dried and deoxygenated on a glass contour system (Pure Process Technology, LLC), stored over 3 Å molecular sieves purchased from Fisher Scientific, and activated prior to use. VO­(OCH_3_)_3_ was synthesized according to a literature precedent.^33^ (Nb­(OC_2_H_5_))_4_)_2_ and [^*n*^Bu_4_N]­[BH_4_] were purchased from Sigma-Aldrich and used as received. POV-alkoxide clusters V_6_O_7_^2–^, V_6_O_7_^1–^, and V_6_O_7_ were prepared according to previously reported procedures.^25,26^ 5,10-Dihydrophenazine was generated following a literature precedent.^57^ Hydrazobenzene was purchased from TCI America and used as received. 1,4-Dihydroxynaphthalene was generated following a literature precedent.^58^

^1^H NMR spectra were recorded at 400 or 500 MHz on a Bruker DPX-400 or Bruker DPX-500 spectrometer, respectively, locked on the signal of deuterated solvents. All chemical shifts were reported relative to the peak of the residual H signal in deuterated solvents. CD_3_CN was purchased from Cambridge Isotope Laboratories, degassed by three freeze–pump–thaw cycles, and stored over fully activated 3 Å molecular sieves. All electrochemistry measurements were performed by using a BioLogic SP-150 potentiostat and acquired with EC-Lab software (V11.42). A glassy carbon disc (3 mm, CH Instruments, USA) and a platinum wire were used as the working and counter electrodes, respectively. A nonaqueous Ag/Ag^+^ reference electrode with 1 mM AgNO_3_ and 100 mM [^*n*^Bu_4_N]­[PF_6_] in acetonitrile (BASi, USA) was used as the reference electrode. All CV measurements were carried out at room temperature in a nitrogen-filled glovebox and calibrated by the Fc^0/+^ couple at 100 mV s^–1^. Infrared (FT-IR, ATR) spectra of the complexes were recorded on a PerkinElmer Spectrum 3 Fourier transform infrared spectrophotometer and are reported in wavenumbers (cm^–1^). Electronic absorption measurements were recorded at room temperature in anhydrous acetonitrile in a sealed 1 cm quartz cuvette with an Agilent Cary 60 UV–vis spectrophotometer or an Agilent Cary 3500 spectrophotometer.

A single crystal of **NbV**_**5**_ **O**_**7**_ was placed onto a nylon loop and mounted on a Rigaku XtaLAB Synergy-S Dualflex diffractometer equipped with a HyPix-6000HE HPC area detector for data collection at 173.00(10) K. A preliminary set of cell constants and an orientation matrix were calculated from a small sampling of reflections.^59^ A short pre-experiment was run, from which an optimal data collection strategy was determined. The full data collection was carried out using a PhotonJet (Cu) X-ray source with frame times of 1.90 and 7.61 s and a detector distance of 34.0 mm. Series of frames were collected in 0.50° steps in *w* at different 2*q*, *k*, and *f* settings. After the intensity data were corrected for absorption, the final cell constants were calculated from the *xyz* centroids of 8422 strong reflections from the actual data collection after integration.^59^ See Table S4 for additional crystal and refinement information. The structure was solved using SHELXT^60^ and refined using SHELXL.^61^ The space group *P*2_1_/*n* was determined based on systematic absences. Most or all non-hydrogen atoms were assigned from the solution. Full-matrix least-squares/difference Fourier cycles were performed, which located any remaining non-hydrogen atoms. All non-hydrogen atoms were refined with anisotropic displacement parameters. All hydrogen atoms were placed in ideal positions and refined as riding atoms with relative isotropic displacement parameters. The final full-matrix least-squares refinement converged to *R*1 = 0.0714 (*F*2, *I* > 2*s*(*I*)) and *wR*2 = 0.1725 (*F*2, all data). The final full-matrix least-squares refinement converged to *R*1 = 0.0433 (*F*2, *I* > 2*s*(*I*)) and *wR*2 = 0.1202 (*F*2, all data).

A single crystal of **NbV**_**5**_ **O**_**6**_ was placed onto a nylon loop and mounted on a Rigaku XtaLAB Synergy-S Dualflex diffractometer equipped with a HyPix-6000HE HPC area detector for data collection at 100.00(10) K. A preliminary set of cell constants and an orientation matrix were calculated from a small sampling of reflections.^59^ A short pre-experiment was run, from which an optimal data collection strategy was determined. The full data collection was carried out using a PhotonJet (Cu) X-ray source with frame times of 0.42 and 1.68 s and a detector distance of 34.0 mm. Series of frames were collected in 0.50° steps in *w* at different 2*q*, *k*, and *f* settings. After the intensity data were corrected for absorption, the final cell constants were calculated from the *xyz* centroids of 35642 strong reflections from the actual data collection after integration.^59^ See Table S1 for additional crystal and refinement information. The structure was solved^60^ using SHELXT2 and refined using SHELXL.^61^ The space group *P*4_3_ was determined based on systematic absences and intensity statistics. Most or all non-hydrogen atoms were assigned from the solution. Full-matrix least-squares/difference Fourier cycles were performed, which located any remaining non-hydrogen atoms. All non-hydrogen atoms were refined with anisotropic displacement parameters. The OH_2_ ligand hydrogen atoms were found from the difference Fourier map and given riding models that preserved their geometry relative to that of the O atom. All other hydrogen atoms were placed in ideal positions and refined as riding atoms with relative isotropic displacement parameters. The final full-matrix least-squares refinement converged to *R*1 = 0.0382 (*F*2, *I* > 2*s*(*I*)) and *wR*2 = 0.0980 (*F*2, all data).

#### Synthesis of [^*n*^Bu_4_N]­[NbV_5_O_7_(OCH_3_)_12_] (**NbV_5_O_7_^1–^**)

In a glovebox, VO­(OCH_3_)_3_ (0.400 g, 2.499 mmol) and [^*n*^Bu_4_N]­[BH_4_] (0.107 g, 0.416 mmol) were added as solids into four 25 mL Teflon-lined autoclave reactors. Nb_2_(OC_2_H_5_)_10_ (0.477 g, 0.750 mmol) was added as a liquid by mass to the reaction mixtures. MeOH (12 mL) was added to each mixture, and they immediately turned dark green as gas evolved. The autoclave reactors were tightly sealed, and the mixtures were heated in an oven at 150 °C for 48 h. The autoclave reactors were allowed to cool to room temperature, after which they were partially unsealed and pumped back into the glovebox. The crude mixtures were combined and filtered over a frit filled with Celite for the removal of a light-brown precipitate. The resulting dark-brown solution was evaporated to dryness under reduced pressure to give a brown solid. The solid was extracted in minimal DCM (∼5 mL) and loaded onto a 60 mL frit half-filled with silica that had been treated with ∼40 mL of DCM. Under a modest vacuum, the frit was repeatedly flushed with DCM (5 × 20 mL) until a brown fraction stopped eluting. This fraction was discarded, and a different 250 mL filter flask was placed under the frit. Then, after repeatedly flushing the frit with THF (5 × 20 mL), the desired green fraction eluted next. Volatiles were removed under vacuum to yield a light-green solid (0.620 g, 0.577 mmol, 35% w.r.t. [^*n*^Bu_4_N]­[BH_4_]). ^1^H NMR (500 MHz, CD_3_CN): δ = 11.3, 24.0, 27.1, 0.97, 1.33, 1.59, 3.07 ppm. FT-IR (ATR, cm^–1^): 1042, 1028 (O–CH_3_), 970, 958 (V=O_t_). UV–vis–NIR (CH_3_CN, 21 °C), 970 nm (ε = 135 M^–1^ cm^–1^), 625 nm (ε = 67 M^–1^ cm^–1^), and 420 nm (ε = 420 M^–1^ cm^–1^). Elemental analysis: Calcd (%) for NbV_5_C_28_H_72_O_19_N (*M*_W_ = 1074.48 g mol^–1^): C, 31.30; H, 6.75; N, 1.30. Found (%): C, 31.48; H, 6.82; N, 1.05.

#### Synthesis of [NbV_5_O_7_(OCH_3_)_12_] (**NbV_5_O_7_**)

In a glovebox, **NbV**_**5**_ **O**_**7**_^**1–**^ (0.150 g, 0.140 mmol) in dichloromethane (5 mL) was prepared in a 20 mL scintillation vial with a stir bar. In the dark, AgOTf (0.040 g, 0.156 mmol) was added using dichloromethane (5 mL). A gray precipitate immediately formed. The mixture of the oxidant and cluster was stirred for 2 h and subsequently filtered over Celite to remove Ag, after which the solvent was removed under reduced pressure. The resulting green solid was extracted with hot pentane (35 °C, 2 × 10 mL), filtered over Celite, and evaporated to dryness to yield **NbV**_**5**_ **O**_**7**_ (0.164 g, 0.197 mmol, 71%). Single crystals of **NbV**_**5**_ **O**_**7**_ suitable for X-ray diffraction were grown from a concentrated solution of pentane. ^1^H NMR (500 MHz, CD_3_CN): δ = 38.50, 14.61, and 9.92 ppm. FT-IR (ATR, cm^–1^): 1023, 1003 (O–CH_3_), 971 (V=O_t_). UV–vis–NIR (CH_3_CN, 21 °C): 387 nm (6617 M^–1^ cm^–1^), and 1000 nm (1036 M^–1^ cm^–1^). Elemental analysis: Calcd (%) for NbV_5_C_12_H_36_O_19_·(0.25 THF) (*M*_W_ = 832.0 g mol^–1^): Calcd (%) C, 18.37; H, 4.51; N, 0.00. Found (%): C, 18.28; H, 4.41; N, 0.00.

#### Synthesis of [NbV_5_O_6_(OH_2_)­(OCH_3_)_12_] (**NbV_5_O_6_**)

In a glovebox, a 20 mL scintillation vial was charged with **NbV**_**5**_ **O**_**7**_ (0.047 g, 0.056 mmol), H_2_Phen (0.010 g, 0.055 mmol), and 10 mL of THF. The reaction mixture immediately changed color from dark green to bright orange. After 1 h, the reaction reached completion, and volatiles were removed under reduced pressure to yield a light-orange solid. The solid was washed with a 3:1 pentane/diethyl ether (4 × 10 mL) until the solution ran clear for the removal of the organic byproduct (Phen). The solid was taken up in THF, filtered over Celite, and extensively dried to yield an isomer mixture of **NbV**_**5**_ **O**_**6**_ (0.039 g, 0.047 mmol, 83%). Single crystals of **NbV**_**5**_ **O**_**6**_ were grown from the slow diffusion of pentane into a solution of THF and a weak base (2,6-lutidine, p*K*_aH_ = 14.16 in MeCN),^62^ with the goal of engaging in a hydrogen-bonding network to facilitate structure ordering; however, no 2,6-lutidine was found to contribute to the obtained structure. ^1^H NMR (500 MHz, CD_3_CN): δ = 30.36, 29.44, 27.16, 26.10, 14.02, 11.13, −10.38, −19.87 ppm. FT-IR (ATR, cm^–1^): 1027 (br) (O–CH_3_), 974, 955 (V=O_t_). Elemental analysis: Calcd (%) for NbV_5_C_12_H_38_O_19_·(CH_2_)_4_O) (*M*_W_ = 906.1 g mol^–1^): C, 21.21; H, 5.12; N, 0.00. Found (%): C, 21.45; H, 4.91; N, 0.00.

#### Purification of *trans*-[NbV_5_O_6_(OH_2_)­(OCH_3_)_12_] (*trans*-**NbV_5_O_6_**)

In a glovebox, NbV_5_O_6_(OH_2_) (0.021 g, 0.025 mmol) was loaded in ∼0.5 mL of THF passed through a silica plug (4:1 THF/diethyl ether eluent). The first fraction was collected, and the solvent was removed under reduced pressure to yield *trans* **-NbV**_**5**_ **O**_**6**_ **(OH**_**2**_ **)** (0.014 g, 0.018 mmol, 70%) (Figure S21). ^1^H NMR (500 MHz, CD_3_CN): δ = 30.36, 14.02, −10.38 ppm. FT-IR (ATR, cm^–1^): 1026 (br) (O–CH_3_), 972, 956 (V=O_t_). UV–vis–NIR (CH_3_CN, 21 °C): 410 nm (323 M^–1^ cm^–1^) and 539 nm (102 M^–1^ cm^–1^). Elemental analysis was not collected for *trans* **-NbV**_**5**_ **O**_**6**_ due to the technique being unable to differentiate between *cis*- and *trans*- isomers. Bulk purity was confirmed by elemental analysis of the isomer mix, **NbV**_**5**_ **O**_**6**_. Isomer purity was assessed and established via ^1^H NMR (Figure S21).

### General Procedure for Thermochemical Analysis of the BDFE­(O–H)_avg_ of NbV_5_O_6_(OH_2_)

Determination of the BDFE­(O–H)_avg_ of **NbV**_**5**_ **O**_**6**_ was performed using reactions between **NbV**_**5**_ **O**_**6**_ and 1,4-dihydroxynaphthalene (H_2_NQ) in THF-*d*_8_. Equimolar amounts of cluster and reductant were measured from stock solutions in THF-*d*_8_, loaded into a J. Young tube, and sealed prior to removal from the glovebox for analysis. Reactions were allowed to react over 10 days at room temperature, with progress tracked by ^1^H NMR spectroscopy (Figures S14 and S15). At this time, the relative concentrations of naphthoquinone (NQ) to H_2_NQ were determined by using the integrations of resonances corresponding with each compound and normalizing for the number of protons each signal represents (Table S3). Upon determination of [H_2_NQ]/[NQ], the adjusted BDFE of the reductant was determined for each reaction using eq 1 (vide infra), where BDFE_H2NQ_ = 62.6 kcal mol^–1^ and *n* = 2, and it is reported as an average of three trials.

$$\mathrm{BDFE}(\mathrm{OH}{)}_{\mathrm{adj}}=\mathrm{BDFE}(\mathrm{OH}{)}_{\mathrm{avg}}-\frac{1.364}{n}\log\;\frac{\left[H_{2}\mathrm{NQ}\right]}{\left[\mathrm{NQ}\right]}$$ 1

### General Procedure for Determining BDFE­(O–H)_avg_ Using DFT

DFT calculations were performed by using the Turbomole 7.3 software package. Geometries were optimized in the gas phase with the PBE0 exchange correlation functional, the def2-TZVP basis set on all atoms, and resolution of the identity approximation (RI-J) with the m4 grid.^63–65^ Dispersion effects were included using the D3 Grimme correction.^66^ Solvent effects were included using the conductor-like screening model (COSMO) solvation model on gas phase optimized geometries.^67,68^ The BDFE­(OH)_avg_ is the average of the bond dissociation free energies calculated from eqs 2 and 3.

$$\begin{array}{l} {\mathrm{NbV}}_{5}O_{7}+H\cdot \to {\mathrm{NbV}}_{5}O_{6}(\mathrm{OH})\\ {\mathrm{BDFE}}_{\left(\mathrm{OH}\right)}=G_{{\mathrm{NbV}}_{5}O_{7}}+G_{H\cdot }-G_{{\mathrm{NbV}}_{5}O_{6}(\mathrm{OH})} \end{array}$$ 2

$$\begin{array}{l} {\mathrm{NbV}}_{5}O_{6}(\mathrm{OH})+H\cdot \to {\mathrm{NbV}}_{5}O_{6}({\mathrm{OH}}_{2})\\ {\mathrm{BDFE}}_{\left({\mathrm{OH}}_{2}\right)}=G_{{\mathrm{NbV}}_{5}O_{6}(\mathrm{OH})}+G_{H\cdot }-G_{{\mathrm{NbV}}_{5}O_{6}({\mathrm{OH}}_{2})} \end{array}$$ 3

## Supplementary Material



## Data Availability

Computational data is available in the following repository: https://doi.org/10.19061/iochem-bd-6-536.

## Notes

Additional spectroscopic data supporting the formation of **NbV**_**5**_ **O**_**7**_^**1–**^, **NbV**_**5**_ **O**_**7**_, and isomers of **NbV**_**5**_ **O**_**6**_ (PDF)
