# Supplementary material for: Nb-Dopant Changes the Regioselectivity of Proton-Coupled Electron Transfer in a Polyoxovanadate-Alkoxide
Source: JACS Au. 2025 Jun 12;5(7):3433–43. doi: 10.1021/jacsau.5c00482 (PMC12308439; doi:10.1021/jacsau.5c00482)
Supplement: Supplementary file 1 [file au5c00482_si_001.pdf]

Electronic Supporting Information

Nb-dopant Changes Regioselectivity of Proton-coupled Electron Transfer in a Polyoxovanadate-alkoxide

Shannon E. Cooney†[a], Thompson V. Marinho†[a], S. Genevieve Duggan[b,c], Dominic Shiels[a], William W. Brennessel[a], Pere Miro[b,c], and Ellen M. Matson*[a]*

[a]*Department of Chemistry, University of Rochester, Rochester, New York 14627, USA*

[b]*Department of Chemistry, University of Iowa, Iowa City, IA 542240, USA*

[c]*Department of Chemistry, University of South Dakota, Vermillion, SD 57069 USA*

† = *authors contributed equally to this work*

Table of Contents of Figures.

Figure S1. top, ESI-MS (negative mode) and bottom, ^{1}H NMR (CD_3CN) of synthetic efforts to access **$NbV_5O_7^{1-}$**. 3
Figure S2. ^{1}H NMR (CD_3CN) of synthetic efforts to access **$NbV_5O_7^{1-}$**. 3
Figure S3. ESI-MS (negative mode) of **$NbV_5O_7^{1-}$**. 4
Figure S4. ^{1}H NMR of top, **$NbV_5O_7^{1-}$** (CD_3CN) compared to bottom, **$V_6O_7^{1-}$**. 4
Figure S5. IR of top, **$V_6O_7^{1-}$** and bottom, **$NbV_5O_7^{1-}$**, neat. 5
Figure S6.Cyclic voltammogram of top, **V_6O_7** and bottom, **$NbV_5O_7^{1-}$**. Inset shows most reduced redox peak of **$NbV_5O_7^{1-}$** with 0.1 M $TBAPF_6$ in CH_3CN. Scan rate 100 mV/s, inset 20 mV/s. 5
Figure S7. **$NbV_5O_7^{1-}$** + H_2Phen, 3 hr 90 °C in CD_3CN. 6
Figure S8. ^{1}H NMR of top, **NbV_5O_7** and bottom, **$NbV_5O_7^{1-}$** in CD_3CN. 6
Figure S9. Cyclic voltammogram of **NbV_5O_7** in CH_3CN in 0.1 M $TBAPF_6$ scan rate 100 mV/s. 7
Figure S10. Crystal structure of **NbV_5O_7**. 7
Figure S11. **NbV_5O_7** + H_2Phen in CD_3CN at RT * indicates the byproduct of the reaction, phenazine. 9
Figure S12. SC XRD structure of **NbV_5O_6** used for bond valence calculations. Vanadium ions used for calculations are labeled with numbers: vacancy V(III) (V_V), V1; equatorial V(IV) (V_e), V2 – V5. 11
Figure S13. **NbV_5O_7** + H_2Azo in CD_3CN at RT.* indicate the byproduct of the reaction, azobenzene. 12
Figure S14. Paramagnetic region of ^{1}H NMR of **NbV_5O_7** + H_2NQ over time in THF-d_8. 13
Figure S15. Diamagnetic region of ^{1}H NMR of **NbV_5O_7** + H_2NQ over time in THF-d_8. 14
Figure S16. Scheme of the equilibrium between **NbV_5O_7** and H_2NQ. 14
Figure S17. EAS of the addition of air to **NbV_5O_6** in THF over the course of 6 hr at RT. 15
Figure S18. Comparison of the reaction of **NbV_5O_7** + H_2Phen in CD_3CN at various temperatures. Top, -30 °C; middle, 23 °C; bottom, 50 °C. 16
Figure S19. Trans-/cis- isomer ratio determined experimentally in situ as a function of dielectric constant using H_2Phen as a reductant. 16
Figure S20. Plots of trans-/cis- isomer ratio as a function of solvent parameter: a. $E_T(30)$, b. donor number, c. acceptor number, d. refractive index, e. viscosity. 17
Figure S21. Comparison of the ^{1}H NMR of isomers of **NbV_5O_6** in CD_3CN. Top, mixture; middle, trans-; bottom, cis-. * indicates residual trans- isomer in the cis- product due to poor margins on the silica gel. 17

Table of Contents of Tables.

Table S1. Crystal data and structure refinement for **NbV_5O_7**. 8
Table S2. Crystal data and structure refinement for **NbV_5O_6**. 10
Table S3. Bond valence calculations for **NbV_5O_6**. 11
Table S4. Structural parameters of **NbV_5O_7** and **TiV_5O_6**. 12
Table S5. Integration of diamagnetic products of the reaction of **NbV_5O_7** + H_2NQ to determine the BDFE(O-H)$_{avg}$ of **NbV_5O_6** from the relative concentrations of species. 15

Link to the DFT calculations repository:

https://doi.org/10.19061/iochem-bd-6-536

References 17

$$Nb_2(OEt)_{10} + 10\ VO(OMe)_3 + 1.7\ {}^{n}Bu_4NBH_4 \xrightarrow[\text{MeOH}]{150\ °C,\ 24hr}$$

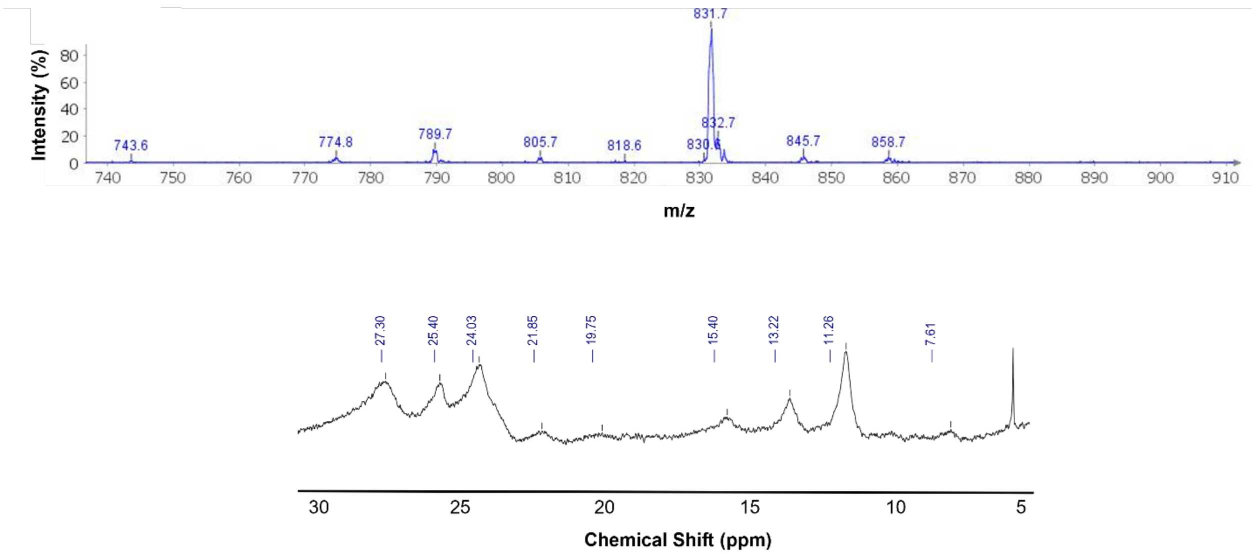

Figure S1. top, ESI-MS (negative mode) and bottom, ^1H NMR (CD_3CN) of synthetic efforts to access $\mathbf{NbV_5O_7^{1-}}$.

$$3\ Nb_2(OEt)_{10} + 10\ VO(OMe)_3 + 2\ {}^{n}Bu_4NBH_4 \xrightarrow[\text{MeOH}]{150\ °C,\ 48hr}$$

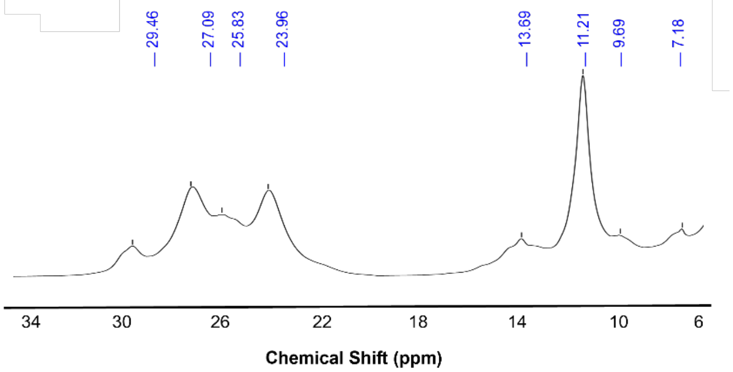

Figure S2. ^1H NMR (CD_3CN) of synthetic efforts to access $\mathbf{NbV_5O_7^{1-}}$.

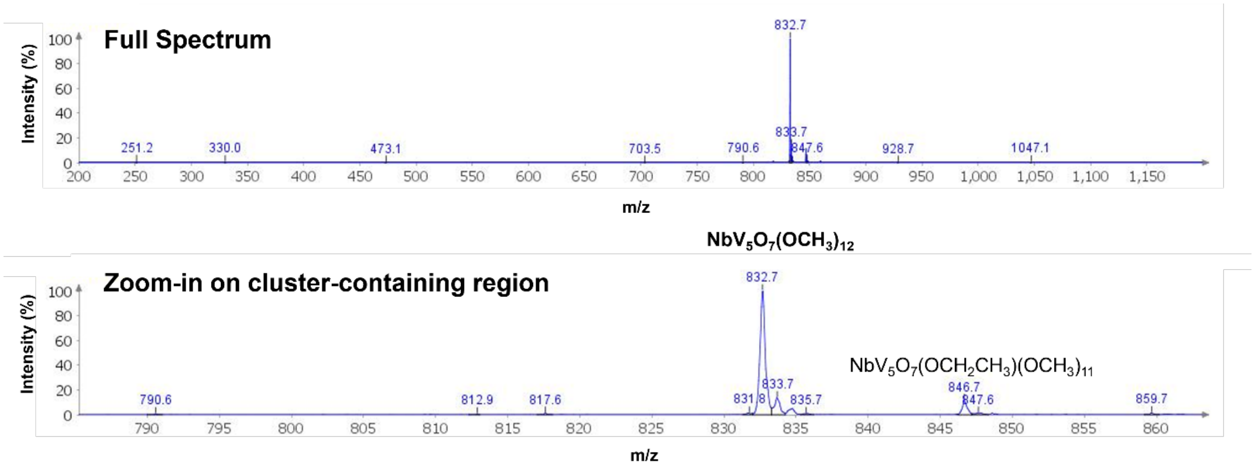

Figure S3. ESI-MS (negative mode) of **$NbV_5O_7^{1-}$**.

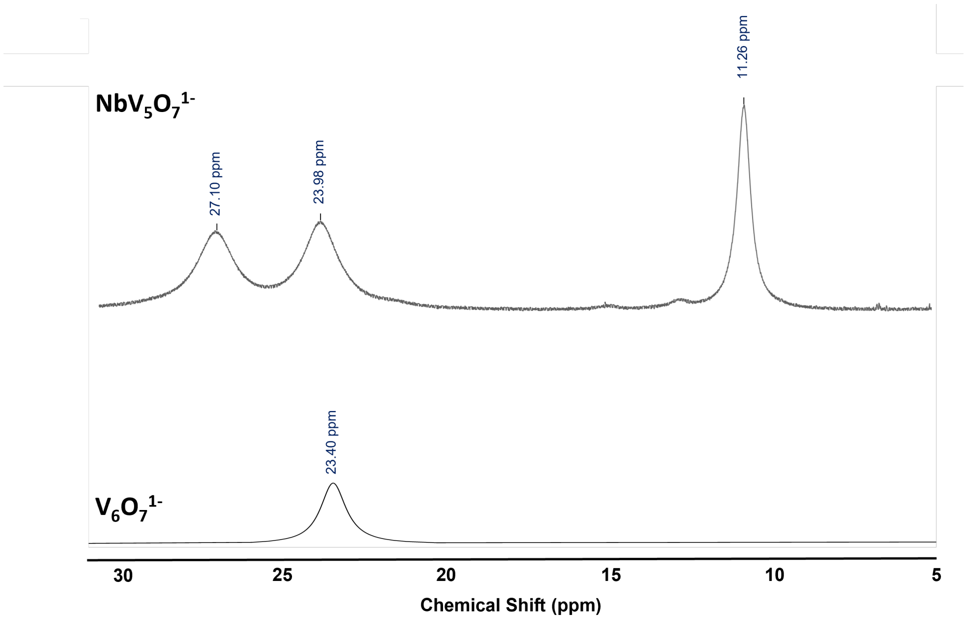

Figure S4. ^{1}H NMR of top, **$NbV_5O_7^{1-}$** (CD_3CN) compared to bottom, **$V_6O_7^{1-}$**.

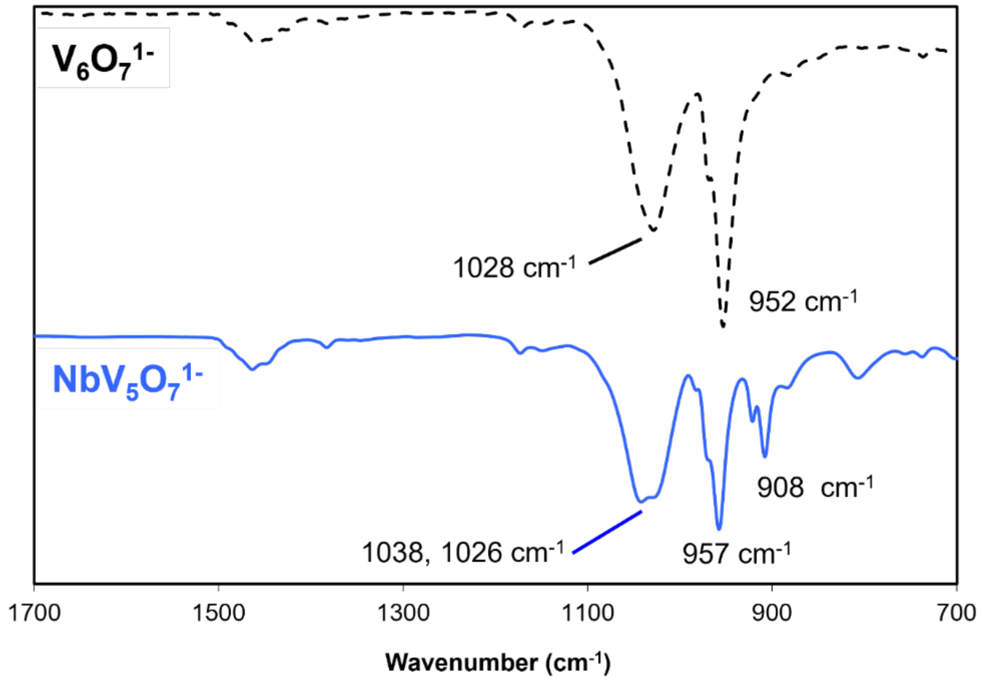

Figure S5. IR of top, $\mathbf{V_6O_7^{1-}}$ and bottom, $\mathbf{NbV_5O_7^{1-}}$, neat.

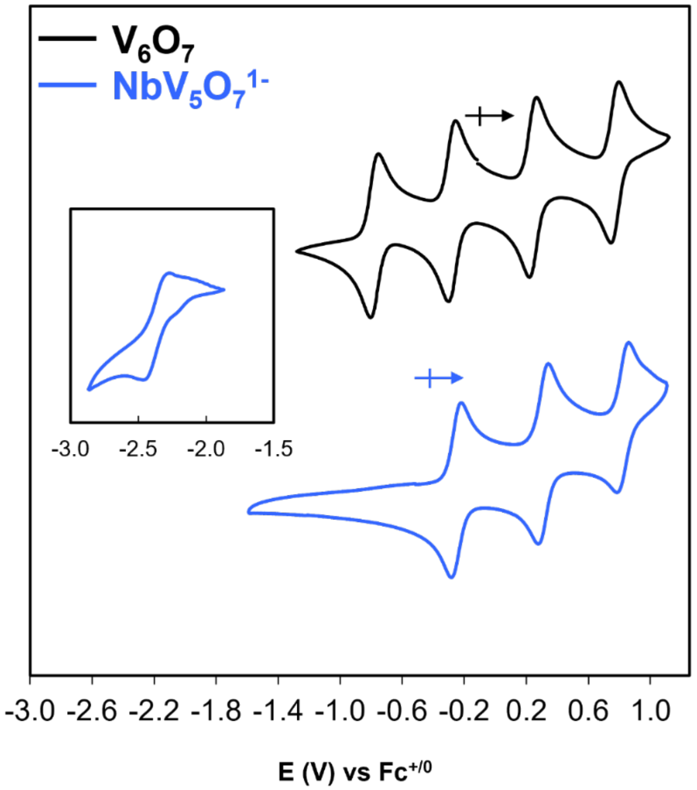

Figure S6.Cyclic voltammogram of top, V_6O_7 and bottom, $\mathbf{NbV_5O_7^{1-}}$. Inset shows most reduced redox peak of $\mathbf{NbV_5O_7^{1-}}$ with 0.1 M $TBAPF_6$ in CH_3CN. Scan rate 100 mV/s, inset 20 mV/s.

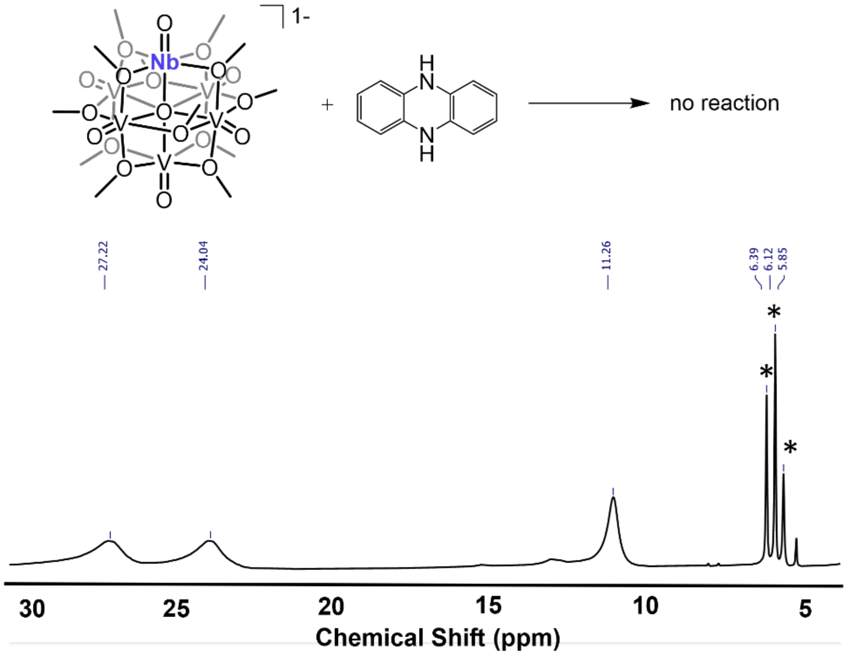

Figure S7. NMR of **$NbV_5O_7$$^{1-}$** + H_2Phen, 3 hr 90 °C in CD_3CN. *denote resonances of unreacted H_2Phen.

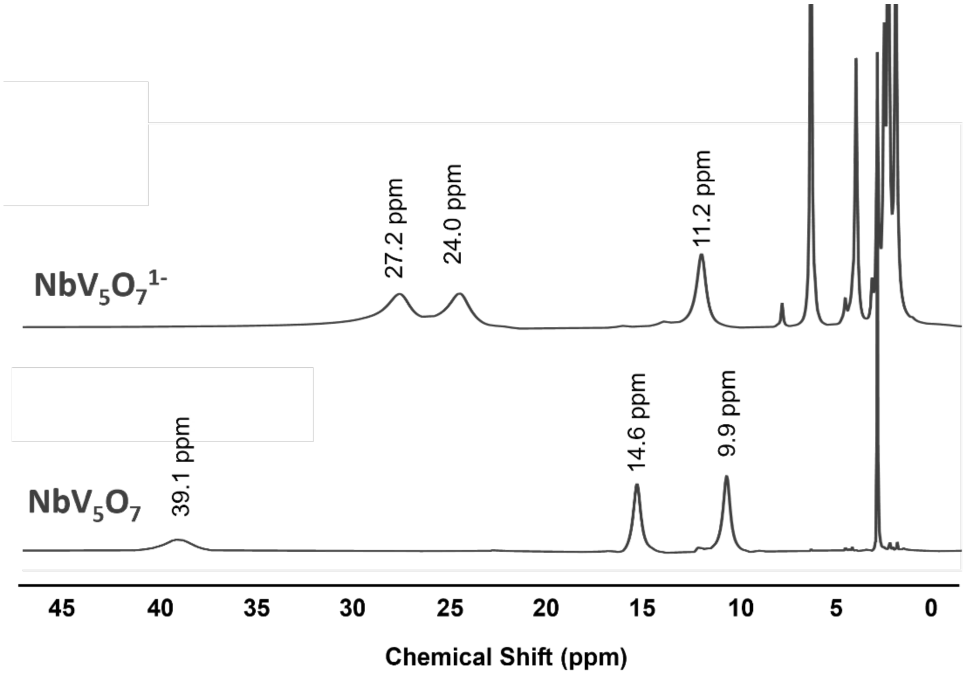

Figure S8. ^{1}H NMR of top, **$NbV_5O_7$$^{1-}$** and bottom, **$NbV_5O_7$** in CD_3CN.

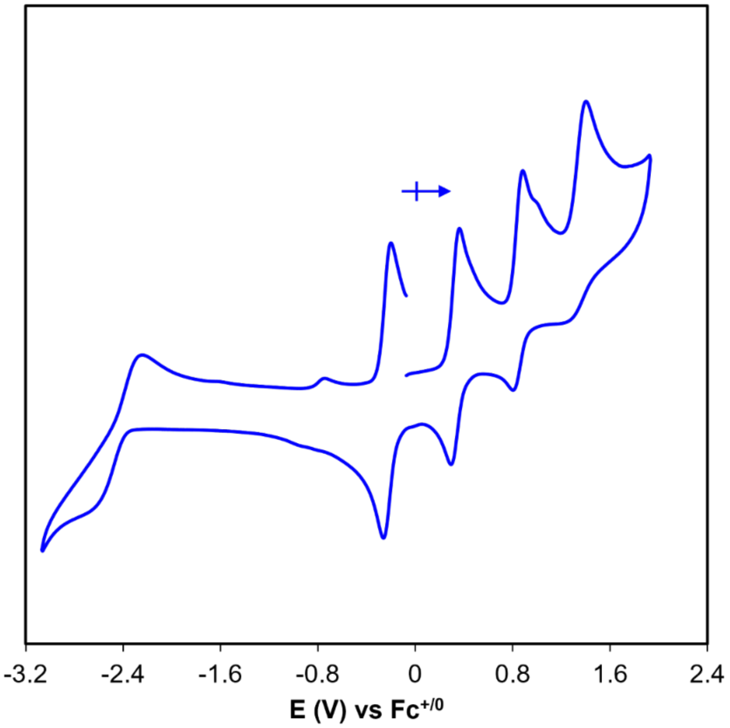

Figure S9. Cyclic voltammogram of $\mathbf{NbV_5O_7}$ in CH_3CN in 0.1 M $TBAPF_6$ scan rate 100 mV/s.

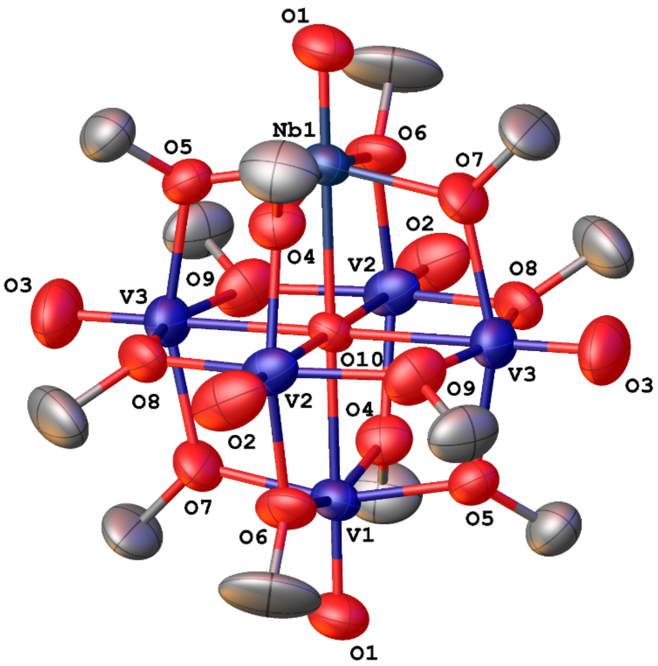

Figure S10. Crystal structure of $\mathbf{NbV_5O_7}$.

Table S1. Crystal data and structure refinement for **NbV_5O_7**.

Identification code	*mattm03; CSD 2419526*	
Empirical formula	*C12 H36 Nb O19 V5*	
Formula weight	*832.02*	
Temperature	*173.00(10) K*	
Wavelength	*1.54184 Å*	
Crystal system	*monoclinic*	
Space group	*$P2_1/n$*	
Unit cell dimensions	*a = 9.2937(5) Å*	*a = 90°*
	b = 9.8119(4) Å	*b = 92.036(5)°*
	c = 15.6762(8) Å	*g = 90°*
Volume	*1428.60(12) $Å^3$*	
Z	*2*	
Density (calculated)	*1.934 Mg/m^3*	
Absorption coefficient	*17.035 mm^{-1}*	
F(000)	*832*	
Crystal color, morphology	*green, plate*	
Crystal size	*0.146 x 0.105 x 0.007 mm^3*	
Theta range for data collection	*5.319 to 82.344°*	
Index ranges	*-11 £ h £ 11, -12 £ k £ 9, -19 £ l £ 19*	
Reflections collected	*18517*	
Independent reflections	*3066 [R(int) = 0.1241]*	
Observed reflections	*2523*	
Completeness to theta = 74.504°	*99.8%*	
Absorption correction	*Multi-scan*	
Max. and min. transmission	*1.00000 and 0.47531*	
Refinement method	*Full-matrix least-squares on F^2*	
Data / restraints / parameters	*3066 / 26 / 189*	
Goodness-of-fit on F^2	*1.065*	
Final R indices [I>2sigma(I)]	*R1 = 0.0714, wR2 = 0.1672*	
R indices (all data)	*R1 = 0.0816, wR2 = 0.1725*	
Largest diff. peak and hole	*0.774 and -0.804 e.$Å^{-3}$*	

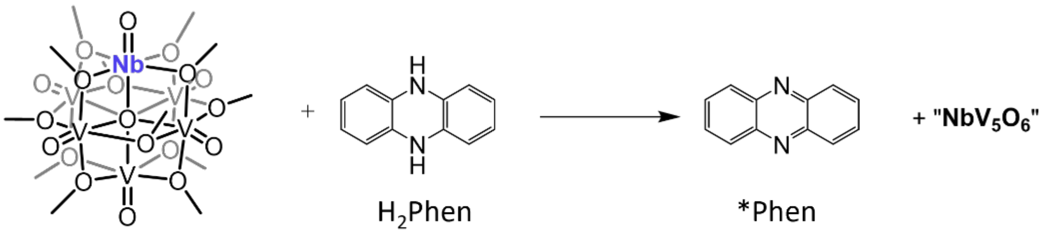

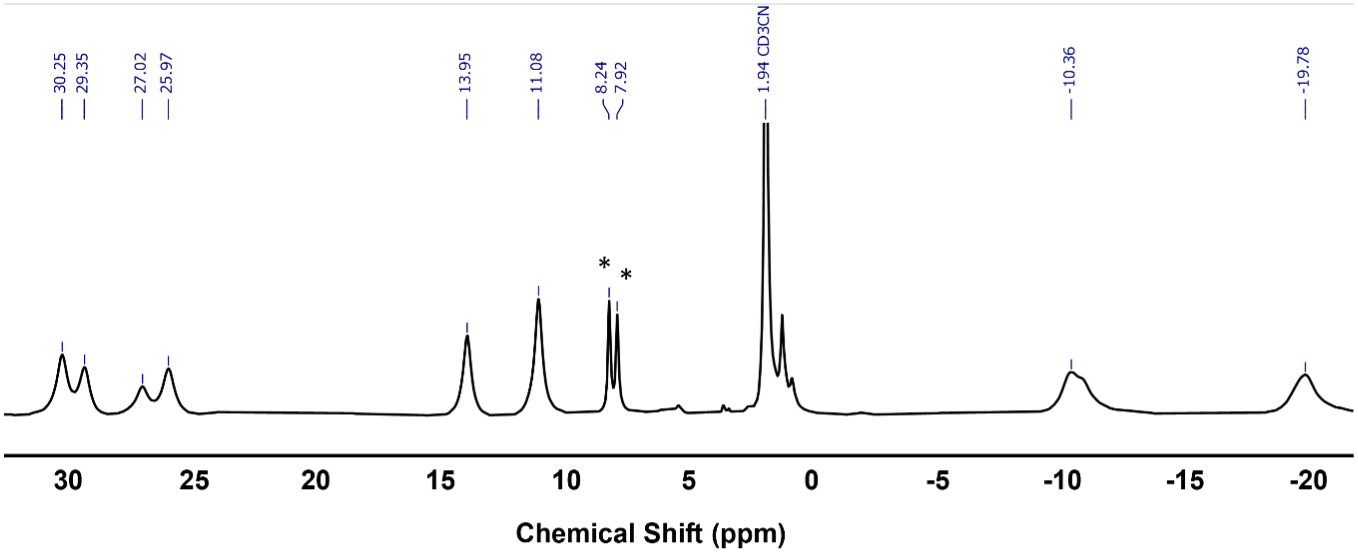

Figure S11. **NbV_5O_7** + H_2Phen in CD_3CN at RT, * indicates the byproduct of the reaction, phenazine.

Table S2. Crystal data and structure refinement for **NbV_5O_6**.

Identification code	*matds13; CSD 2419278*	
Empirical formula	*C20 H54 Nb O21 V5*	
Formula weight	*978.24*	
Temperature	*100.00(10) K*	
Wavelength	*1.54184 Å*	
Crystal system	*tetragonal*	
Space group	$P4_3$	
Unit cell dimensions	*a = 10.47482(6) Å*	*a = 90°*
	b = 10.47482(6) Å	*b = 90°*
	c = 33.5448(2) Å	*g = 90°*
Volume	*3680.60(5)* $Å^3$	
Z	*4*	
Density (calculated)	*1.765* Mg/m^3	
Absorption coefficient	*13.369* mm^{-1}	
F(000)	*1992*	
Crystal color, morphology	*orange-brown, needle*	
Crystal size	*0.424 x 0.066 x 0.049* mm^3	
Theta range for data collection	*4.221 to 80.123°*	
Index ranges	*-13 £ h £ 13, -13 £ k £ 12, -42 £ l £ 42*	
Reflections collected	*61989*	
Independent reflections	*7939 [R(int) = 0.0587]*	
Observed reflections	*7713*	
Completeness to theta = 67.684°	*100.0%*	
Absorption correction	*Multi-scan*	
Max. and min. transmission	*1.00000 and 0.06023*	
Refinement method	*Full-matrix least-squares on* F^2	
Data / restraints / parameters	*7939 / 207 / 563*	
Goodness-of-fit on F^2	*1.020*	
Final R indices [I>2sigma(I)]	*R1 = 0.0382, wR2 = 0.0974*	
R indices (all data)	*R1 = 0.0392, wR2 = 0.0980*	
Absolute structure parameter	*0.001(5)*	
Largest diff. peak and hole	*0.789 and -0.407* $e.Å^{-3}$	

Table S3. Bond valence calculations for **NbV_5O_6**.

	V1 (V_V)	*V2 (V_e)*	*V3 (V_e)*	*V4 (V_e)*	*V5 (V_e)*
V(III)	***3.08***	*3.854*	*3.747*	*3.928*	*3.948*
V(IV)	*3.143*	***3.963***	***3.836***	***4.021***	***4.042***
V(V)	*3.379*	*4.200*	*4.096*	*4.286*	*4.307*

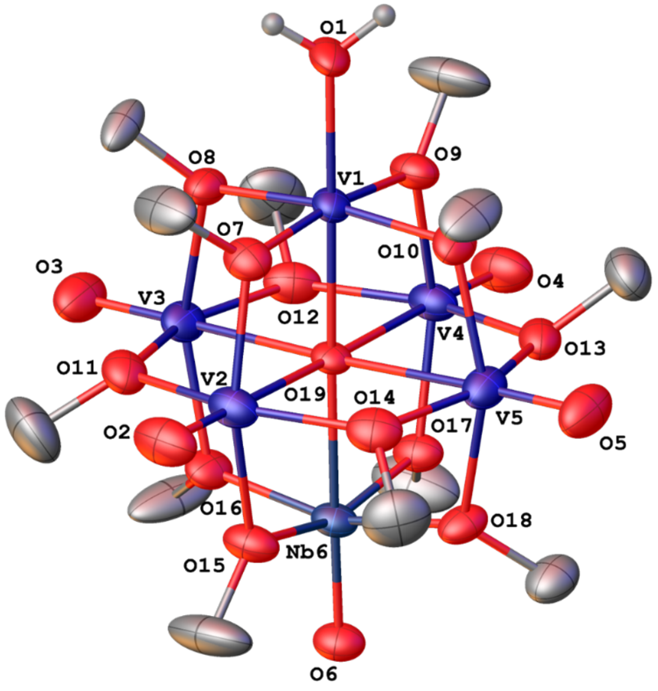

Figure S12. SC XRD structure of **NbV_5O_6** used for bond valence calculations. Vanadium ions used for calculations are labeled with numbers: vacancy V(III) (V_V), V1; equatorial V(IV) (V_e), V2 – V5.

Table S4. Structural parameters of **NbV_5O_7** and **TiV_5O_6**.[1]

Bond	NbV_5O_7	TiV_5O_6
$M–O_t$	1.611(5) Å	1.771(7) Å
$M–O_c$	2.3121(9) Å	2.030(5) Å
$V_a–O_c$	2.3121(9) Å	2.500(5) Å
V_a-O_t	1.611(5) Å	1.583(6) Å
$V_e–O_c$ (avg)	2.3243 Å	2.3343(1) Å
$V_e–O_t$ (avg)	1.605 Å	1.592(5) Å

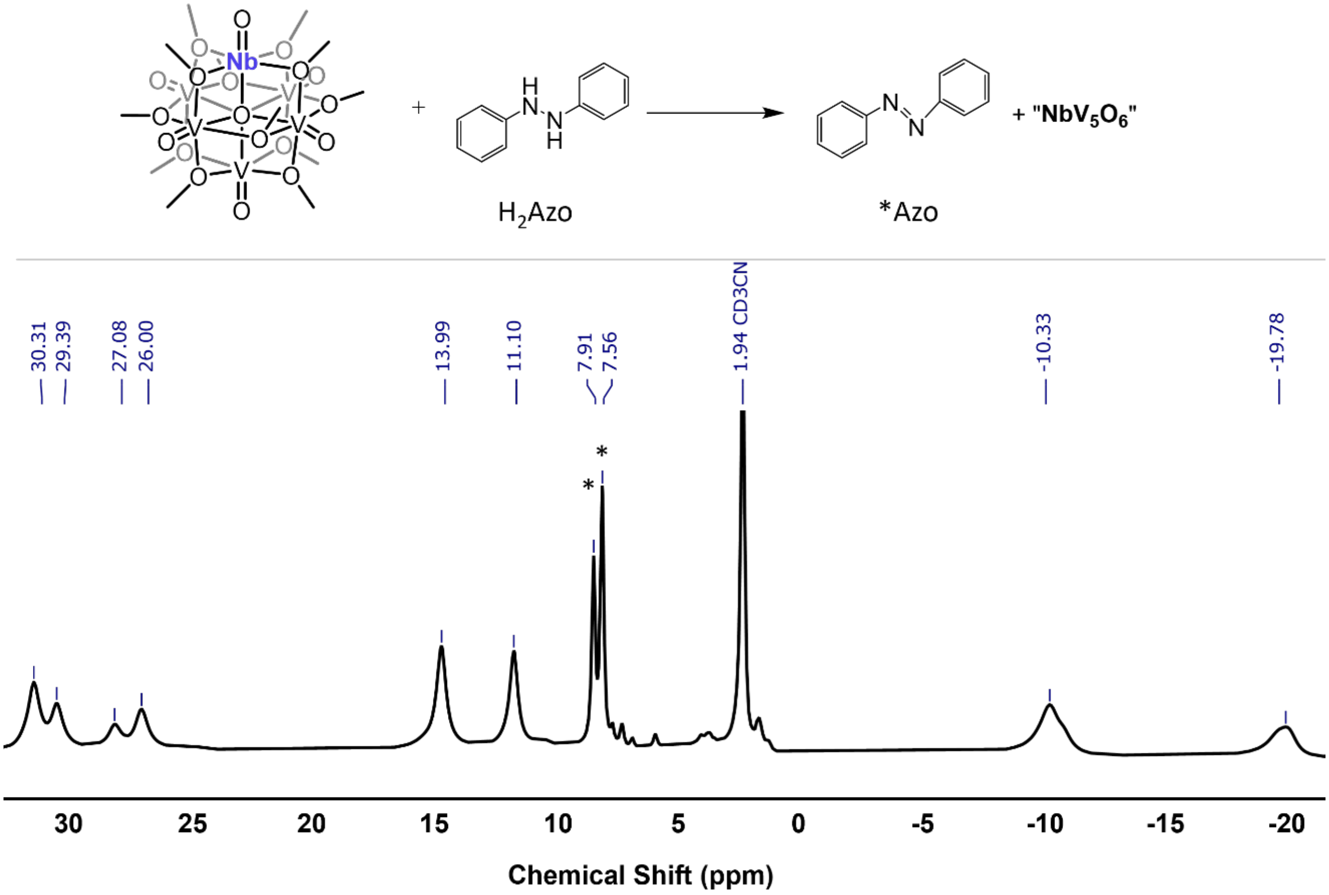

Figure S13. **NbV_5O_7** + H_2Azo in CD_3CN at RT.* indicate the byproduct of the reaction, azobenzene.

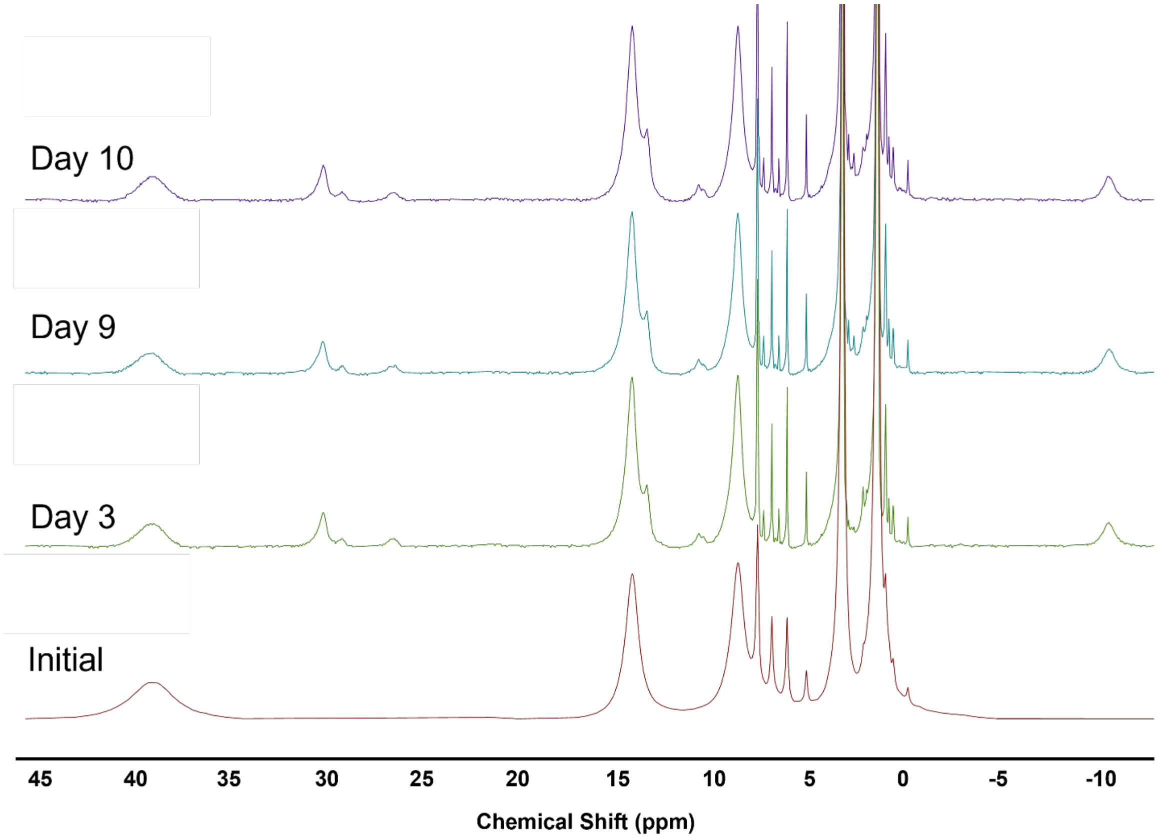

Figure S14. Paramagnetic region of ^{1}H NMR of **NbV_5O_7** + H_2NQ over time in THF-d_8.

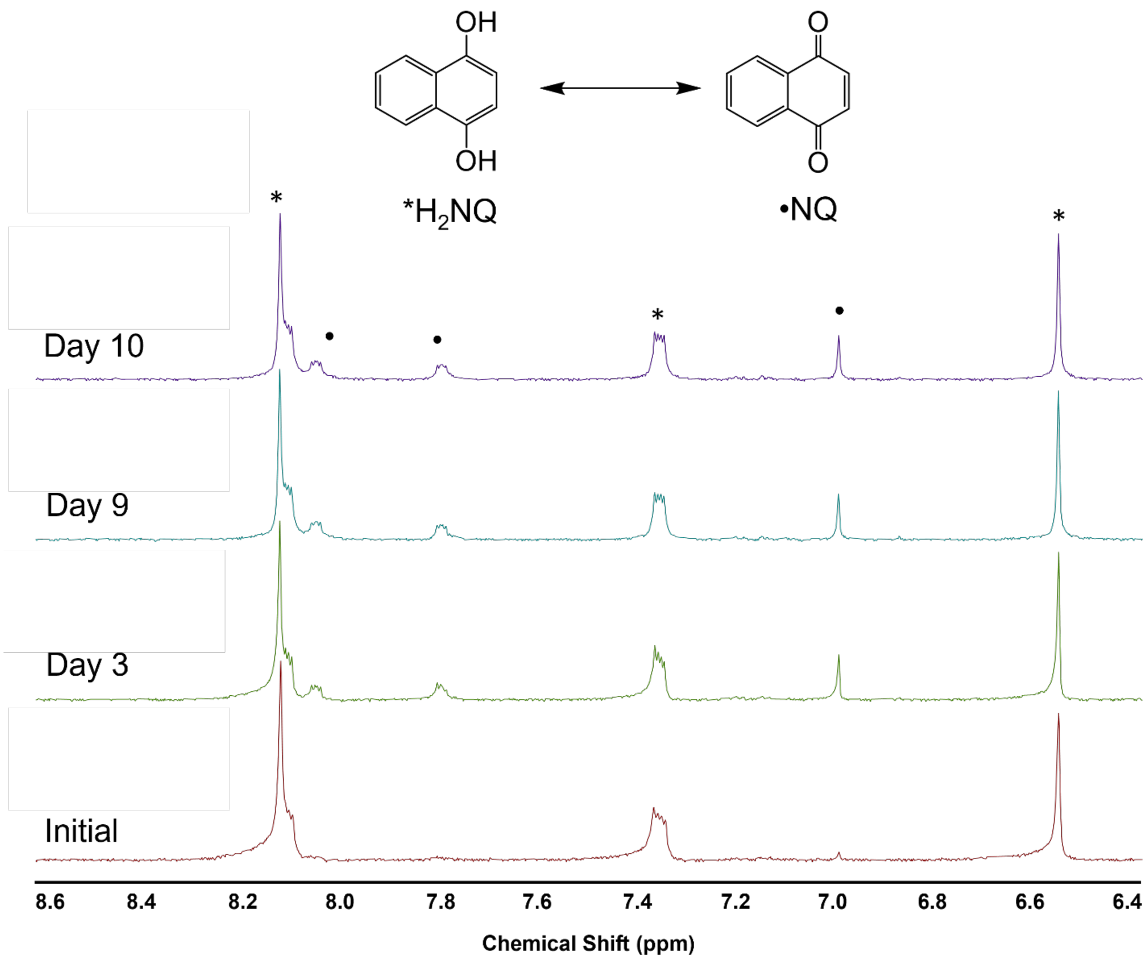

Figure S15. Diamagnetic region of ^{1}H NMR of **NbV_5O_7** + H_2NQ over time in THF-d_8.* denotes starting material, H_2NQ and • denotes formation of the dehydrogenated product, NQ.

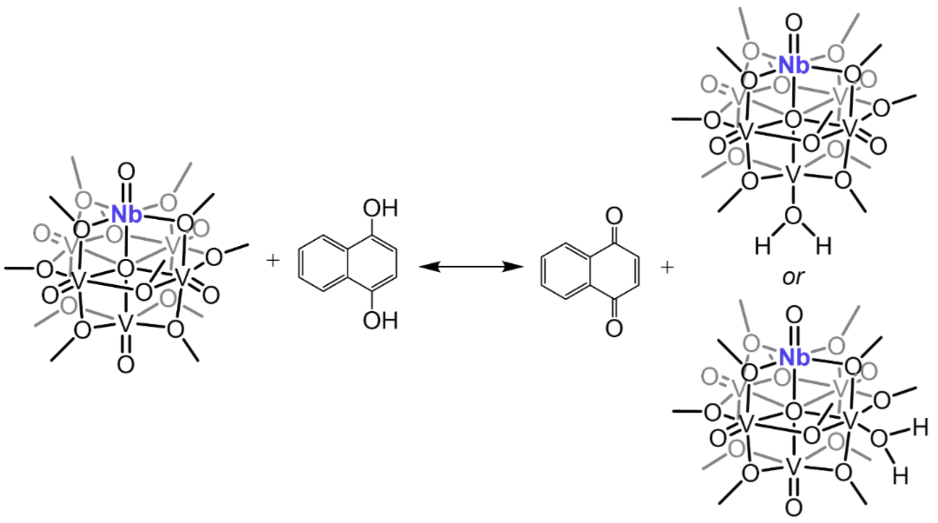

Figure S16. Scheme of the equilibrium between **NbV_5O_7** and H_2NQ.

Table S5. Integration of diamagnetic products of the reaction of **NbV_5O_7** + H_2NQ to determine the BDFE(O-H)$_{avg}$ of **NbV_5O_6** from the relative concentrations of species. Integration (Int) of either H_2NQ or NQ. Relative concentration (Rel) is the integration divided by the number of protons associated with the peak. Average (avg) is the average relative concentration between two proton signals of the organic complex. The ratio of reduced to oxidized species ($[H_2NQ]/[NQ]$) is determined from the average relative concentrations. Triplicate trials presented.

	**1,4 -H_2NQ**					**NQ**						
	8.1 ppm (2H)		7.45 ppm (2H)			7.83 ppm (2H)		7.0 ppm (2H)				
Trial	Int.	Rel	Int.	Rel	Avg	Int.	Rel	Int.	Rel	Avg	$[H_2NQ]/[NQ]$	BDFE
A	3.15	1.58	2.96	1.48	1.53	1	0.5	1.05	0.525	0.51	2.98	62.3
B	3.36	1.68	3.36	1.68	1.68	1	0.5	0.83	0.42	0.46	3.67	62.2
C	3.50	1.75	3.71	1.86	1.80	1	0.5	0.87	0.44	0.47	3.86	62.2
											Average	**62.3 kcal/mol**
											St dev	0.04

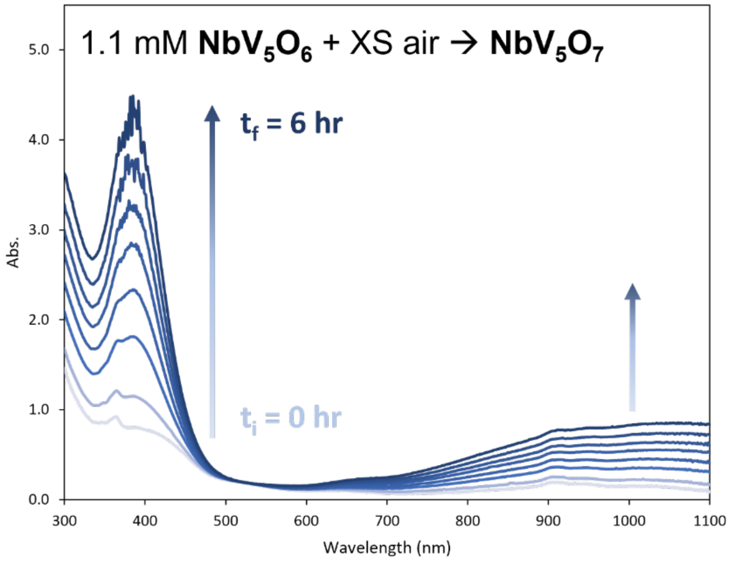

Figure S17. EAS of the addition of air to **NbV_5O_6** in THF over the course of 6 hr at RT.

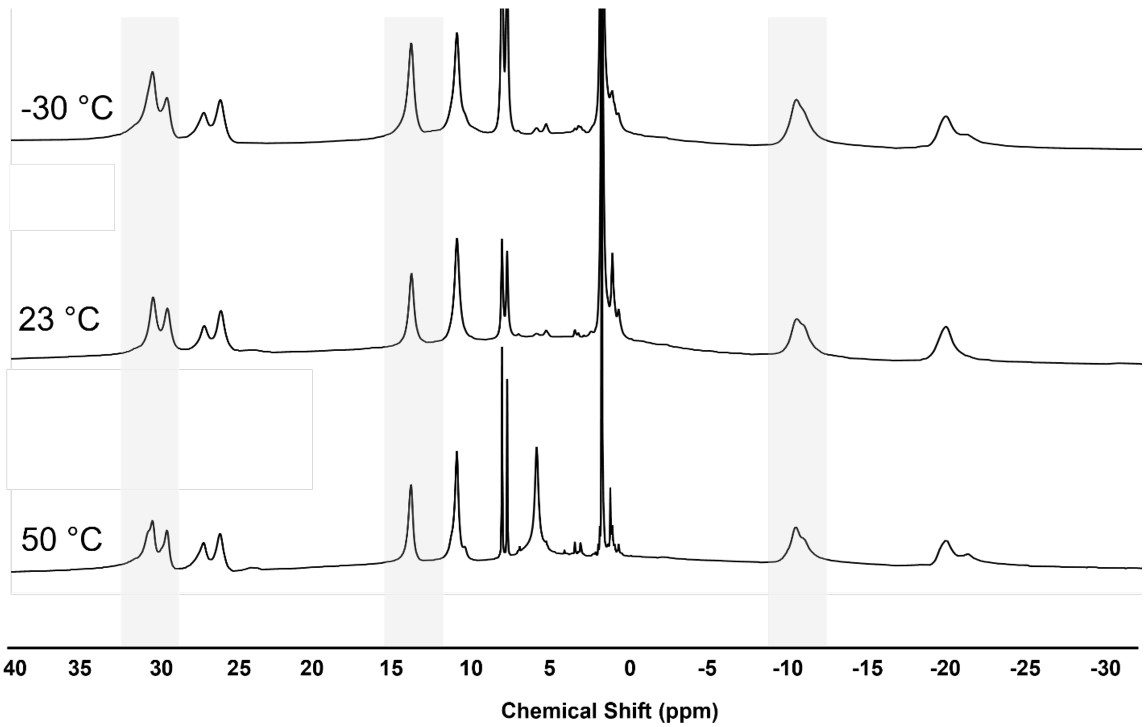

Figure S18. Comparison of the reaction of **NbV_5O_7** + H_2Phen in CD_3CN at various temperatures. Top, -30 °C; middle, 23 °C; bottom, 50 °C. Peaks associated with *trans*-$NbV_5O_6(OH_2)$ are highlighted in blue.

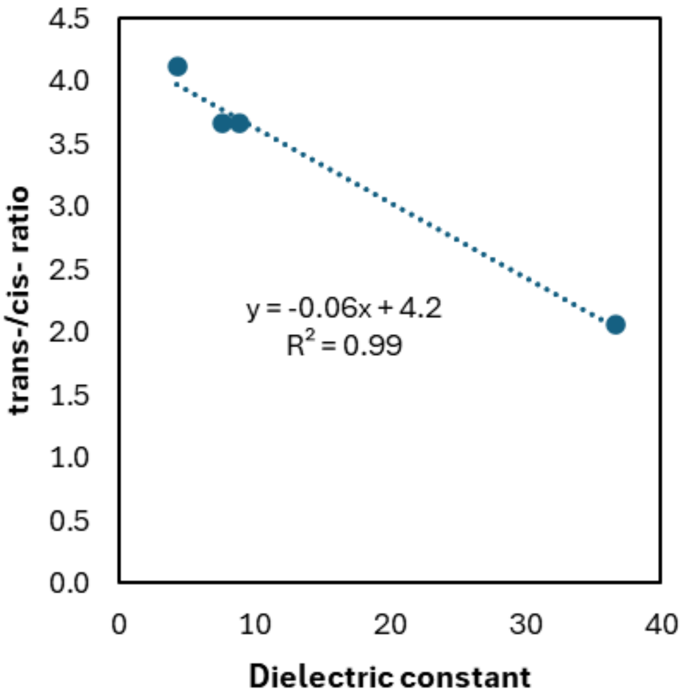

Figure S19. *Trans-*/*cis-* isomer ratio determined experimentally *in situ* as a function of dielectric constant using H_2Phen as a reductant.

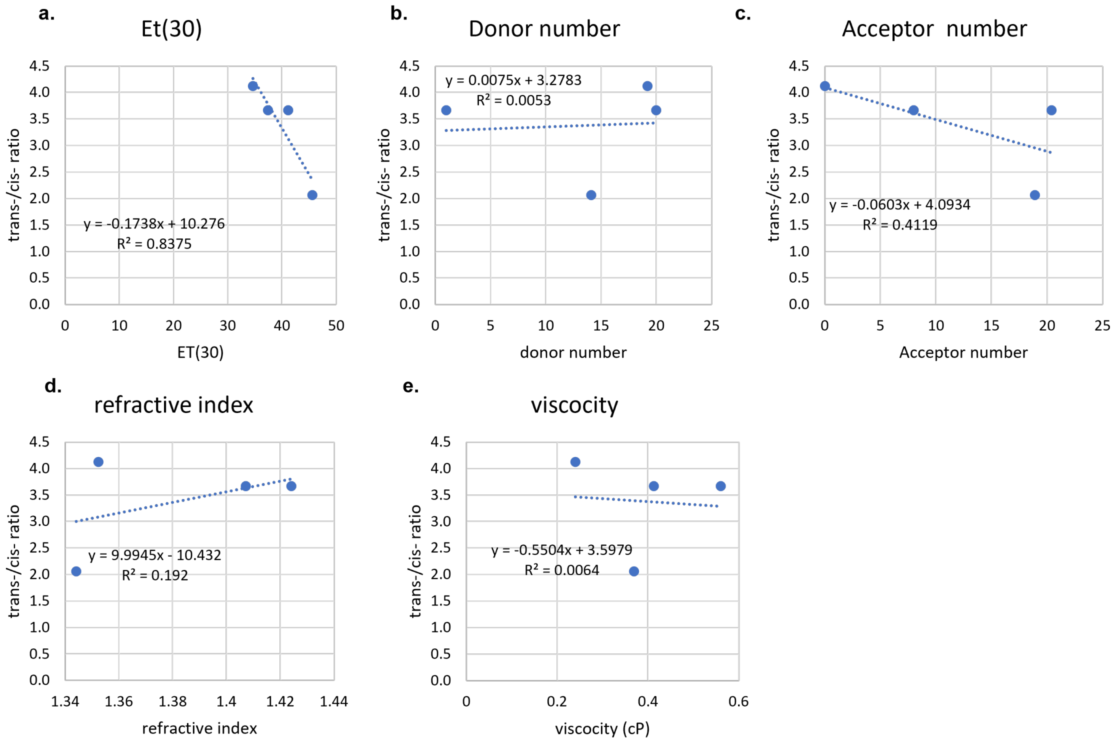

Figure S20. Plots of *trans-/cis-* isomer ratio as a function of solvent parameter: a. E_T(30), b. donor number, c. acceptor number, d. refractive index, e. viscosity.

.

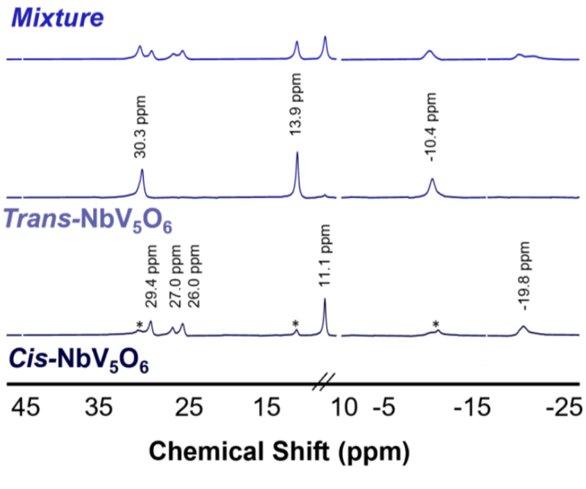

Figure S21. Comparison of the ^{1}H NMR of isomers of **NbV_5O_6** in CD_3CN. Top, mixture; middle, *trans*-; bottom, *cis*-. * indicates residual *trans*- isomer in the *cis*- product due to poor margins on the silica gel.

References.

1. Cooney, S. E.; Walls, M. R. A.; Schreiber, E.; Brennessel, W. W.; Matson, E. M. *J. Am. Chem. Soc.* **2024**. *146*, 4, 2364 – 2369.
